# Highly Efficient and Mild Gold (I) Catalyzed Synthesis of 3,8-Diarylidene-2,7-dioxaspiro[4.4]nonane-1,6-diones

**DOI:** 10.3390/molecules28010300

**Published:** 2022-12-30

**Authors:** Antonia Iazzetti, Dario Allevi, Andrea Calcaterra, Giancarlo Fabrizi, Antonella Goggiamani, Giulia Mazzoccanti, Alessio Sferrazza, Rosanna Verdiglione, Valeria Vergine

**Affiliations:** 1 Dipartimento di Scienze Biotecnologiche di Base Cliniche Intensivologiche e Perioperatorie, Università Cattolica del Sacro Cuore, Campus di Roma, Largo Francesco Vito 1, 00168 Rome, Italy; 2 Department of Chemistry and Technology of Drugs, Sapienza-University of Rome, P.le Aldo Moro 5, 00185 Rome, Italy

**Keywords:** spirobislactone, spirolactone, spiro compounds, Au(I)-catalyzed cyclization, alkyne activation, heterocyclization

## Abstract

The gold-catalyzed cyclization of 2,2-bis(3-arylprop-2-yn1-yl)malonic acid has been proposed as an efficient approach to substituted 3,8-dibenzyl-2,7-dioxaspiro[4.4]nonane-1,6-diones. The reaction proceeds smoothly in mild reaction conditions to give the desired products in quantitative yields in the presence of variously substituted starting materials. In addition, the synthesis of γ-arylidene spirobislactone bearing different substituents on the two aromatic rings has been achieved. This kind of compound could be of great interest in pharmaceutical science given the widespread presence of this scaffold in bioactive natural and synthetic products.

## 1. Introduction

**Figure 1:**
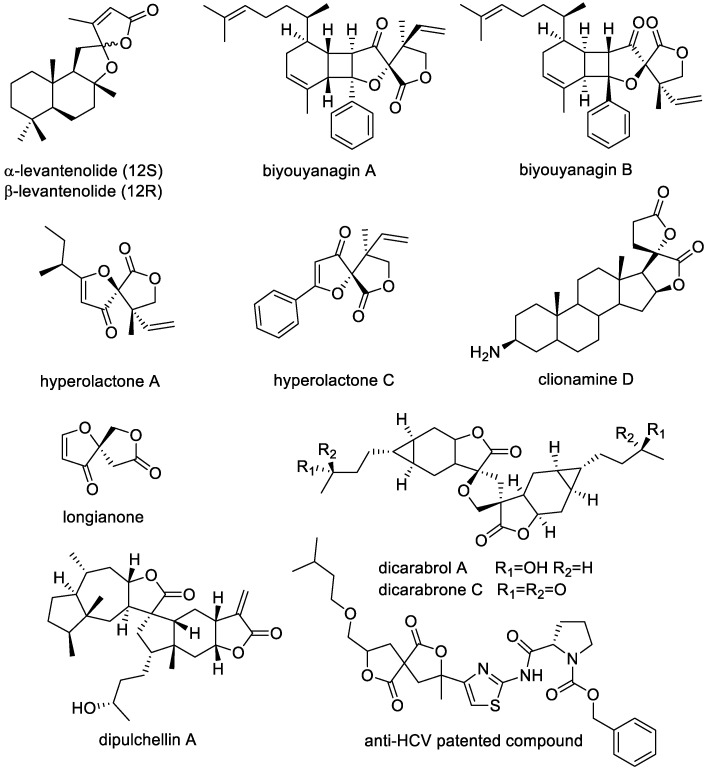
Bioactive natural and synthetic products containing spirobislactone moiety or similar scaffold.

3,8-dibenzyl-2,7-dioxaspiro[4.4]nonane-1,6-diones are molecules endowed with a complex γ-benzyl-spirobis-γ-lactone scaffold, which gained a great interest from researchers due to their biological activity and appealing conformational features, which include the presence of an element of chirality, namely a stereocenter or a chirality axis. A great number of unique bioactive natural products are endowed with a spirolactone moiety [1]. Among these, some compounds contain a spirobislactone unit or a very similar moiety, as α- and β-levantenolides, biyouyanagin A and B, hyperolactones A and C, clionamide D, longianone (Figure 1) [2]. Moreover, some natural products isolated from *Carpesium abrotanoides* bearing a similar γ-dilactones scaffold, namely dicarabrol A, dicarabrone C, and dipulchellin A (Figure 1), showed promising biological activities, including anti-inflammatory, antitumor, antiplasmodial, and bactericidal effects [3]. In 2007 a patent reported, among the others, a compound bearing a spirodilactone moiety (Figure 1) with antiviral activity against *Flaviviridae* family of viruses, in particular HCV [4]. More recently, a granted patent describes the preparation and use of spirobislactones as useful crosslink reagents for the synthesis of polymeric materials to be employed in coating processes for automotive applications [5]. Considering the preparation of spirobislactones, the first synthetic routes have been established by simple methods, using multistep synthesis that includes the cyclization of the two spirolactone rings in separate reaction steps, with low overall yields [6,7,8,9].

Roland et al. reported in 1963 the preparation of spirolactones by the reaction of dibromomalononitrile with two equivalents of ethylene, followed by acid hydrolysis [10]. Kotha et al. afforded spirobislactone scaffold by treating diethyl diallylmalonate with sulfuric acid [11]. The diseleno-derivative of spirobislactone was obtained by a Japanese group as a mixture of diastereoisomers by treating diethyl diallylmalonate with phenylselenyl chloride [12,13]. Maldemy et al. reported the cyclization of malonate derivatives with iodine(III) reagents to afford the spirobislactone [14]. The use of acid, base, and metal catalysis played a key role in the synthetic access to spirobislactones. Bronsted acids as triflic acid or concentrated sulfuric acid have been used in the dilactonization of *tert*-butyl diallylmalonate [15], and diethyl diallylmalonate, respectively [11]. Fairlamb et al. discovered that the treatment of dimethyl diallylmalonate with the Lewis acid SbF_5_ generated from HSbF_6_ during a Pd catalyzed cycloisomerization of 1,6-dienes, afforded instead of the desired monocyclized product a mixture of stereoisomer of the dimethyl spirobislactone [16]. Only one example in literature reported the use of polymer-supported bases like PS-DMAP or PS-BEMP to catalyze the spirodilactonization of phenyl glycidyl ether with dimethyl malonate, in a solvent-free reaction [17]. The use of metal catalysts is without any doubt highly efficient for the spirodilactonization process, starting from acyclic precursors. In 1980s two different groups reported an oxidative spirodilactonization reaction of malonic acid in presence of an alkene catalyzed by Mn(III) acetate [18,19,20]. Copper (II) or silver (I) triflates were used by Adrio et al. [21] and Yang et al. [22], respectively, to afford dilactonization of diallymalonic acid to the spirobislactone scaffold, by intramolecular addition of carboxylic acid to the olefin moiety, which selectively led to a spiro-γ-bislactone.

**Scheme 1:**
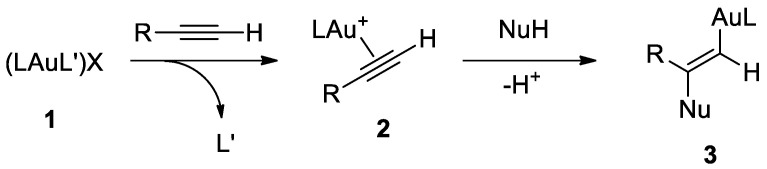
Activation of alkynes by electrophilic Au(I) complexes and nucleophilic addition to the C-C triple bond. A labile ligand (L′) is exchanged with the alkyne, forming a π-complex (**2**), followed by the anti-addition of a nucleophile to the η^2^-[AuL]^+^-activated alkyne (**2**).

Recently, the use of gold (I) catalysts for the activation of C-C triple bond opened new horizons for the construction of complex molecular scaffolds [23], including for the synthetic access to spirobislactones starting from dipropargylmalonate derivatives. As shown by the wide range of substrate and product scope reported by a recent review [23], gold(I)-catalyzed activation of alkynes is a consolidated method for the construction of molecular complexity. The first examples of gold(I) activation of alkynes were reported by Teles [24] and Tanaka [25], demonstrating the potential of gold(I) in organic synthesis. Currently, gold(I) complexes are the most efficient catalysts for the electrophilic activation of alkynes, and the formation of new carbon−carbon or carbon−heteroatom bonds can be achieved by using different nucleophiles [26,27,28]. Au(I) complexes usually are linear and two-coordinate, as highlighted by a significant number of alkyne-gold complexes that have been characterized and studied in solution or theoretically. Generally, the most effective catalysts for the activation of alkynes are complexes [LAuL′]X or [LAuX], which contain weakly coordinating neutral (L′) or anionic ligand (X^−^). These complexes can enter catalytic cycles by ligand (often a nitrile) exchange with the unsaturated substrate. Gold(I) complexes selectively activate π-bonds of alkynes in complex way, which has been explained by relativistic effects. Generally, the nucleophilic Markovnikov attack to η^2^-[AuL]^+^-activated alkynes **1** forms trans-alkenyl-gold complexes **2** as intermediates (Scheme 1) [23].

In this field, we recently investigated the gold-catalyzed intramolecular hydroarylation reaction of *N*-ethoxycarbonyl-*N*-propargylanilines as effective tool for the preparation of 4-substituted-1,2-dihydroquinolines [29], and the synthesis of polycyclic chromene cores through gold (I)-catalyzed intramolecular hydroarylation reaction [30]. Based on these studies, we evaluated the possibility to obtain a gold (I) synthetic approach to spirobislactones. This class of compounds exhibit remarkable features which justify a continue efforts in the development of synthetic procedures to access them. Additionally, at the best of our knowledge, only few examples of gold(I) catalysis successfully employed for the preparation of spirobislactones have been reported in literature. In 2013, Chiarucci et al. reported the synthesis of γ-vinylbutyrolactones by intramolecular oxaallylic alkylation with alcohols. Starting from dimethyl 2,2-bis((*E*)-4-hydroxybut-2-en-1-yl)malonate they afforded the corresponding γ-methylenespirobislactone by using IMesAuCl/AgOTf as catalyst [31]. A similar γ,γ′-dibenzylspirobislactone was obtained by Zhu et al. by gold-catalyzed homogeneous oxidative carboheterofunctionalization of diallylmalonic acid, in the presence of phenylboronic acid, SIMesAuCl as catalytst, and selectfluor as an oxidant [32]. Gold nanoparticles stabilized by PEG-tagged imidazolium salts have also been used as recyclable catalysts for the cycloisomerization of γ-alkynoic acids into enol-lactones, among the other products, a γ,γ-divinylspirobislactone was obtained starting from dipropargylmalonic acid [33].

Finally, spirobislactones could be prepared as single enantiomers by using chiral Au(I) complexes, as recently reported by Lin et al. [34]. The application of phosphine-nitrogen chiral ligands and related gold (I) complexes used as catalytic systems for the asymmetric reactions have been patented in Chine from the same group [35].

**Scheme 2:**
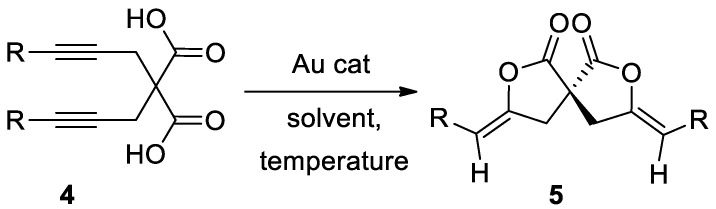
Work hypothesis.

In this paper are reported the results of our studies that led to the development of a method for the synthesis of spirolactones **5** starting from substituted dialkynylmalonic acids **4** (Scheme 2).

## 2. Results and Discussion

**Figure 2:**
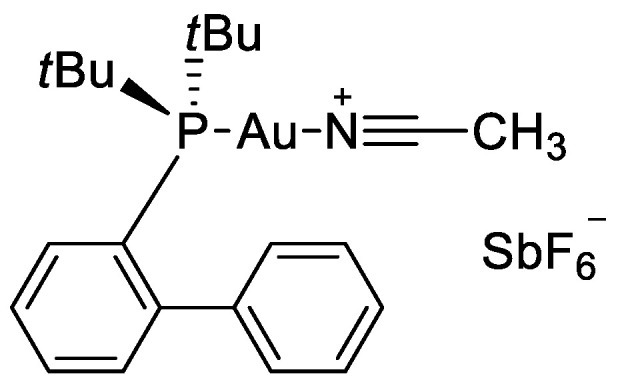
Chemical structure of JohnPhosAu(MeCN)SbF_6_ catalyst.

To start our work, we decided to investigated the Au-catalyzed cyclization of 2,2-bis(arylprop-2-ynyl)malonic acids **4** affording 3,8-diarylidene-2,7-dioxaspiro[4.4]nonane-1,6-diones **5** (Table 1). Our preliminary experiments to establish the best reaction conditions for the conversion of 2,2-bis(3-phenylprop-2-ynyl)malonic acid **4a** into the corresponding spirolactone **5a** are reported in the following Table 1. No cyclization was observed in presence of palladium catalyst or in presence of Au(III) (Table 1, entry 1,2). In contrast, the use of an electrophilic complex of Au(I) with the Buchwald’s sterically bulky biaryl phosphine ligand JohnPhos and acetonitrile as catalyst (Figure 2), led the formation of the desired product in quantitative yields, and in very mild conditions (Table 1, entry 5). Indeed, the best results were obtained at room temperature, very likely due to the decrease of decomposition reactions.

**Scheme 3:**
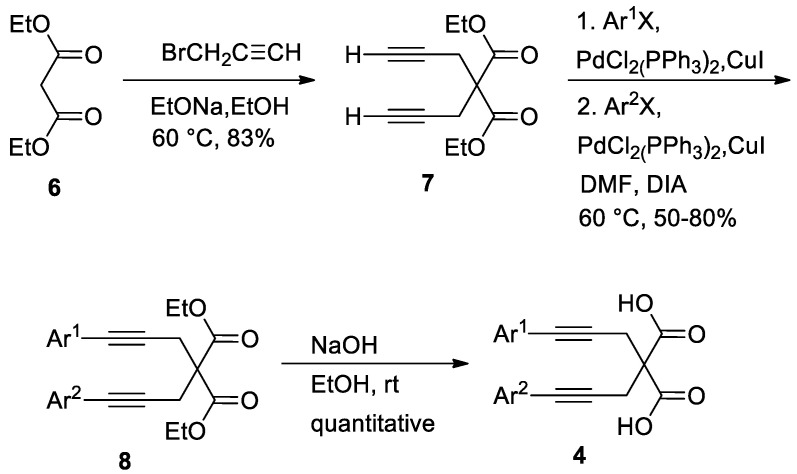
Synthesis of symmetrical or unsymmetrical diethyl diarylalkynylmalonates **8** and diarylalkynylmalonic acids **4**.

The reaction scope was investigated by using other 2,2-bis(3-arylprop-2-ynyl)malonic acids (**4**) (Table 2) in the same conditions optimized for substrate **4a**. 2,2-bis(3-arylprop-2-ynyl)malonic acids **4** used for these studies were prepared by alkylation of diethyl malonate **6** with propargyl bromide, followed by two Sonogashira cross-coupling with two different aryl halides. Then, the hydrolysis of functionalized diethyl malonate completes the sequences for the preparation of symmetrical or unsymmetrical substrates **4** to be investigated in the gold-catalyzed spirodilactonization reaction (Scheme 3). Since the substituted malonic acid **4a** was obtained in a quantitative yield from the last step, the hydrolysis reaction of the other esters **8** was then performed in sequence with the Au (I)-promoted cyclization (Table 2), without isolating the acids **4**.

Under the optimized reaction conditions several derivatives bearing a variety of useful functional groups have been prepared and obtained in a quantitative yield. The synthesized compounds are summarized in Table 2.

## 3. Materials and Methods

### 3.1. General Experimental Procedures

Melting points were recorded with a Büchi melting point B-545 and are not corrected. ^1^H and ^13^C NMR spectra have been acquired with a Bruker Avance 400 spectrometer operating at 400.13 and 100.6 MHz, respectively, at 300 K in DMSO-d_6_, using 5 mm diameter glass tubes. Chemical shifts were expressed in ppm and coupling constants (*J*) in hertz (Hz), approximated to 0.1 Hz. The residual solvent peak was used as an internal reference for ^1^H and ^13^C NMR spectra. Data for ^1^H NMR are reported as follows: chemical shift, multiplicity (br = broad, ovrlp = overlapped, s = singlet, d = doublet, t = triplet, q = quartet, m = multiplet, dd = double doublet), coupling constant, integral. Spectra were processed with the program MestReNova version 6.0.2–5475, FT and zero filling at 64 K. High-resolution (HR) mass spectra were obtained using a Thermo Fischer Exactive mass spectrometer equipped with an ESI source and an Orbitrap analyzer: capillary temperature 275 °C, spray voltage 3.5 kV, sheath gas (N_2_) 10 arbitrary units, capillary voltage 65 V, tube lens 125 V. Analytical TLC was performed using 0.25 mm Fluka F254 silica gel. The compounds on TLC were revealed by quenching fluorescence (at 254 or 365 nm) using a 4W UV lamp. Otherwise, TLC plates were stained with *p*-anisaldehyde acidic solution in EtOH or a 10% solution of phosphomolybdic acid in EtOH and heated (T = 120 °C). The product mixture purifications were carried out with silica column chromatography using Fluka 60 Å silica gel (0063–0200 mm, 70–230 mesh). Flash chromatography was performed using 200−400 mesh silica gel. Commercially available reagents and solvents were supplied by Sigma-Aldrich (St. Louis, MI, USA) and used without further purification.

### 3.2. Procedure for the Screening of Optimal Catalyst, Temperature and Additive for the Gold (I)-Catalyzed Synthesis of 3,8-Dimethylene-2,7-dioxaspiro[4.4]nonane-1,6-diones ***5***

In a 5 mL Carousel Tube Reactor (Radley Discovery Technology) containing a magnetic stirring bar Au(I) catalyst (0.02 mmol) was dissolved in 1.0 mL of CH_2_Cl_2_. Then, 2,2-di(prop-2-ynyl)malonic acid (0.5 mmol) was added and the reaction mixture was stirred for a time ranging from 0.5 to 24 h at a temperature ranging from room temperature to 100 °C. After this time, *n*-hexane was added to the reaction mixture and then it was centrifuged. The precipitate was separated from the liquid layer to obtain 3,8-dimethylene-2,7-dioxaspiro[4.4]nonane-1,6-dione.

### 3.3. Procedures for the Synthesis of Stating Materials

Diethyl 2,2-di(prop-2-yl)malonate (**7**) has been synthetized according to known procedures and their characterization data match our own in all respects [36,37].

**diethyl 2,2-di(prop-2-yn-1-yl)malonate (7)**: colorless oil, 83%. ^1^H NMR (400 MHz, CDCl_3_) δ 4.21 (q, *J* = 7.1 Hz, 4H), 2.96 (d, *J* = 2.6 Hz, 4H), 2.01 (m, 2H), 1.24 (t, *J* = 7.1 Hz, 6H). ^13^C NMR (CDCl_3_): δ 168.7, 78.5, 71.8, 62.2, 56.4, 22.6, 14.1.

### 3.4. Typical Procedure for the Preparation of 2,2-Bis(3-phenylprop-2-yn-1-yl)malonate *(****8a****)*

Compounds **8a, b, c, f, g, h, i, j, k, l, n** have been prepared according to the typical procedure described below, by using suitable aryl iodides.

A flask equipped with a magnetic stirring bar was charged with PdCl_2_(PPh_3_)_2_ (42 mg, 0.06 mmol, 0.02 equiv.) and CuI (22.8 mg, 0.12 mmol, 0.04 equiv.) dissolved in diisopropylamine (12 mL) and *N*,*N*-dimethylformamide (6 mL). The resultant solution was stirred under nitrogen at room temperature for 10 min before adding iodobenzene (1346 mg, 736 μL, 6.6 mmol, 2.2 equiv.) and diethyl 2,2-di(prop-2-yn-1-yl)malonate (**7**) (709 mg, 3.0 mmol, 1.0 equiv.) and stirred for 4 h at room temperature. After this time, the reaction mixture was diluted with Et_2_O and washed with a saturated NH_4_Cl solution, HCl 2 N, and with brine. The organic layer was dried over Na_2_SO_4_, filtered, and concentrated under reduced pressure. The residue was purified by chromatography on SiO_2_ (25–40 μm), eluting with an 80/20 (*v*/*v*) *n*-hexane/AcOEt mixture to obtain 1106 mg (90% yield) of diethyl 2,2-bis(3-phenylprop-2-yn-1-yl)malonate **8a**.

### 3.5. Typical Procedure for the Preparation of Diethyl 2-(3-(3-methoxyphenyl)prop-2-yn-1-yl)-2-(3-phenylprop-2-yn-1-yl)malonate *(****8d****)*

Compounds **8d**, **e**, **m** were prepared according to the typical procedure described below, by using suitable aryl iodides.

A flask equipped with a magnetic stirring bar was charged with a solution of PdCl_2_(PPh_3_)_2_ (42 mg, 0.06 mmol, 0.02 equiv.) and CuI (22.8 mg, 0.12 mmol, 0.04 equiv.) in diisopropylamine (12 mL) and *N,N*-dimethylformamide (6 mL), iodobenzene (673 mg, 368 μL, 3.3 mmol, 1.1 equiv.) and diethyl 2,2-di(prop-2-yn-1-yl)malonate (**7**) (709 mg, 3.0 mmol, 1.0 equiv.). The reaction mixture was stirred at room temperature under argon for 4 h before adding 1-iodo-3-methoxybenzene (772 mg, 3.3 mmol, 1.1 equiv.). After 6 h, the mixture was diluted with Et_2_O and washed with a saturated NH_4_Cl solution, HCl 2 N, and with brine. The organic layer was dried over Na_2_SO_4_, filtered, and concentrated under reduced pressure. The residue was purified by chromatography on SiO_2_ (25–40 μm), eluting with an 80/20 (*v*/*v*) *n*-hexane/AcOEt mixture to obtain 628 mg (50% yield) of 2-(3-(3-methoxyphenyl)prop-2-yn-1-yl)-2-(3-phenylprop-2-yn-1-yl)malonate (**8d**).

**diethyl 2,2-bis(3-phenylprop-2-yn-1-yl)malonate (8a):** yellow pale oil. ^1^H NMR (400 MHz, CDCl3) δ 7.44–7.37 (m, 4H), 7.30 (m, 6H), 4.30 (q, *J* = 7.1 Hz, 4H), 3.30 (s, 4H), 1.31 (t, *J* = 7.1 Hz, 8H). ^13^C NMR (101 MHz, CDCl_3_) δ 169.0, 131.8, 128.3, 128.1, 123.2, 84.2, 83.8, 62.1, 57.2, 23.8, 14.2.

**diethyl 2,2-bis(3-(4-methoxyphenyl)prop-2-yn-1-yl)malonate (8c):** yellow pale oil. ^1^H NMR (CDCl_3_): δ 7.33 (d, *J* = 8.3 Hz, 4H), 6.82 (d, *J* = 8.3 Hz, 4H), 4.28 (q, *J* = 7.0 Hz, 4H), 3.81 (s, 6H), 3.25 (s, 4H), 1.30 (t, *J* = 7.0 Hz, 6H). ^13^C NMR (CDCl_3_): δ 169.2, 159.4, 133.1, 115.4, 113.9, 83.5, 82.7, 62.0, 57.3, 55.4, 23.8, 14.2.

**diethyl 2-(3-(4-methoxyphenyl)prop-2-yn-1-yl)-2-(3-phenylprop-2-yn-1-yl)malonate (8d):** yellow pale oil. ^1^H NMR (CDCl_3_): δ 7.45–7.36 (m, 2H), 7.31 (m, 1H), 7.21 (t, *J* = 7.9 Hz, 1H), 7.01 (d, *J* = 7.4 Hz, 1H), 6.94 (br s, 1H), 6.87 (m, 1H), 4.30 (q, *J* = 7.1 Hz, 4H), 3.81 (s, 3H), 3.29 (s, 4H), 1.31 (t, *J* = 7.2 Hz, 6H). ^13^C NMR (CDCl_3_): δ 169.0, 159.4, 131.8, 129.4, 128.3, 128.1, 124.4, 124.3, 123.2, 116.7, 114.6, 84.2, 84.1, 83.8, 83.7, 62.1, 57.2, 55.3, 29.8, 23.8, 14.2.

**diethyl 2,2-bis(3-(4-methylphenyl)prop-2-yn-1-yl)malonate (8f):** yellow pale oil. ^1^H NMR (400 MHz, CDCl_3_): δ 7.30 (d, *J* = 8.0 Hz, 4H), 7.10 (d, *J* = 7.8 Hz, 4H), 4.29 (q, *J* = 7.1 Hz, 4H), 3.28 (s, 4H), 2.35 (s, 6H), 1.30 (t, *J* = 7.1 Hz, 6H). ^13^C NMR (CDCl_3_): δ 169.0, 138.1, 131.6, 129.0, 120.2, 83.8, 83.5, 62.0, 60.4, 57.3, 23.8, 21.5, 14.2.

**diethyl 2,2-bis(3-(naphthalen-1-yl)prop-2-yn-1-yl)malonate (8g):** yellow pale oil. ^1^H NMR (CDCl_3_): δ 8.34 (d, *J* = 8.2 Hz, 2H), 7.82 (dd, *J* = 12.2, 8.2 Hz, 6H), 7.65 (d, *J* = 7.0 Hz, 2H), 7.60–7.46 (m, 6H), 7.40 (t, *J* = 7.7 Hz, 2H), 4.33 (q, *J* = 7.1 Hz, 4H), 3.54 (s, 4H), 1.31 (t, *J* = 7.1 Hz, 6H). ^13^C NMR (CDCl_3_): δ 169.3, 133.6, 133.3, 130.7, 128.6, 128.4, 126.9, 126.5, 126.4, 125.3, 121.0, 89.2, 82.0, 62.3, 57.3, 24.5, 14.3.

**diethyl 2,2-bis(3-(4-bromo-3-fluorophenyl)prop-2-yn-1-yl)malonate (8i):** yellow pale oil. ^1^H NMR (CDCl_3_): δ 7.47 (t, *J* = 7.7 Hz, 1H), 7.13 (d, *J* = 9.1 Hz, 1H), 7.05 (d, *J* = 8.2 Hz, 1H), 4.29 (q, *J* = 7.1 Hz, 2H), 3.23 (s, 2H), 1.30 (t, *J* = 7.1 Hz, 3H). ^13^C NMR (CDCl_3_): δ 168.8, 160.0, 157.5, 133.5, 128.7, 128.7, 124.2, 124.1, 119.7, 119.5, 109.6, 109.4, 86.3, 82.1, 62.3, 56.9, 23.9, 14.3.

**diethyl 2,2-bis(3-(4-chlorophenyl)prop-2-yn-1-yl)malonate (8j)**: yellow pale oil. ^1^H NMR (CDCl_3_): δ 7.32 (d, *J* = 8.6 Hz, 4H), 7.29–7.23 (m, 4H), 4.29 (q, *J* = 7.1 Hz, 4H), 3.25 (s, 4H), 1.30 (t, *J* = 7.1 Hz, 6H). ^13^C NMR (CDCl_3_): δ 168.9, 134.2, 133.0, 128.7, 121.7, 85.3, 82.8, 62.2, 57.1, 23.9, 14.2.

**diethyl 2,2-bis(3-(4-bromophenyl)prop-2-yn-1-yl)malonate (8k)**: yellow pale oil. ^1^H NMR (CDCl_3_): δ 7.43 (d, *J* = 8.2 Hz, 4H), 7.26 (t, *J* = 8.8 Hz, 4H), 4.28 (q, *J* = 7.1 Hz, 4H), 3.25 (s, 4H), 1.29 (t, *J* = 7.0 Hz, 6H). ^13^C NMR (CDCl_3_): δ 168.9, 139.2, 133.6, 133.2, 131.6, 122.4, 122.1, 85.5, 82.9, 62.2, 57.0, 23.9, 14.2.

**diethyl 2,2-bis(3-(2-methyl-5-nitrophenyl)prop-2-yn-1-yl)malonate (8l):** yellow pale oil. ^1^H NMR (CDCl_3_): δ 8.18 (d, *J* = 1.8 Hz, 2H), 8.02 (dd, *J* = 8.4, 1.9 Hz, 2H), 7.33 (d, *J* = 8.4 Hz, 2H), 4.28 (q, *J* = 7.1 Hz, 4H), 3.34 (s, 4H), 2.49 (s, 6H), 1.29 (t, *J* = 7.1 Hz, 6H). ^13^C NMR (CDCl_3_): δ 168.7, 147.8, 146.1, 130.3, 127.0, 124.4, 122.9, 90.6, 80.8, 62.4, 56.7, 24.1, 21.1, 14.2.

**diethyl 2-(3-(4-chlorophenyl)prop-2-yn-1-yl)-2-(3-(4-methoxyphenyl)prop-2-yn-1-yl)malonate (8m):** yellow pale oil. ^1^H NMR (CDCl_3_): δ 7.30 (d, *J* = 3.6 Hz, 2H), 7.28 (d, *J* = 3.6 Hz, 3H), 7.23 (d, *J* = 8.5 Hz, 3H), 6.78 (d, *J* = 8.7 Hz, 2H), 4.25 (q, *J* = 7.1 Hz, 4H), 3.77 (s, 3H), 3.22 (d, *J* = 9.1 Hz, 4H), 1.32–1.19 (*J* = 7.1 Hz, 6H). ^13^C NMR (CDCl_3_): δ 169.0, 159.5, 134.1, 133.1, 133.0, 128.6, 121.8, 115.3, 113.9, 85.5, 83.7, 82.7, 82.5, 62.1, 57.2, 55.3, 23.9, 23.8, 14.2.

**diethyl 2,2-bis(3-(2-methoxyphenyl)prop-2-yn-1-yl)malonate (8n):** yellow pale oil. ^1^H NMR (CDCl_3_): δ 7.33 (dd, *J* = 7.5, 1.4 Hz, 2H), 7.25–7.20 (m, 2H), 6.86–6.81 (m, 4H), 4.24 (q, *J* = 7.1 Hz, 4H), 3.83 (s, 6H), 3.33 (s, 4H), 1.26 (t, *J* = 7.1 Hz, 6H). ^13^C NMR (CDCl_3_): δ 169.1, 160.2, 133.8, 129.4, 120.4, 112.7, 110.8, 88.7, 79.9, 62.0, 57.5, 55.8, 24.1, 14.2.

### 3.6. General Procedure for the Synthesis of 3,8-Dimethylenespiro[4.4]nonane-1,6-diones *(****5****)*

In a 10 mL round bottom flask, 2,2 disubstituted diethyl malonate (**8**) (0.5 mmol) was dissolved in 2 mL of EtOH and 1 mL of NaOH 2N was added. The reaction mixture was heated at reflux and stirred for one hour. After cooling, the reaction mixture was acidified with conc. HCl, diluted with Et_2_O, and washed with brine. The organic layer was dried over Na_2_SO_4_, filtered, and concentrated under reduced pressure. The residue was used without further purification in the next step. In a 5 mL Carousel Tube Reactor (Radley Discovery Technology) containing a magnetic stirring bar JohnPhos Au(MeCN)SbF_6_ catalyst (0.02 mmol) was dissolved at room temperature in 1.0 mL of CH_2_Cl_2_. Then, 2,2-disubstituted malonic acid (0.5 mmol) was added and the reaction mixture was stirred for 1 h at room temperature. After this time, *n*-hexane was added to the reaction mixture and then it was centrifuged. The precipitate was separated from the liquid layer to obtain 3,8-dimethylene-2,7-dioxaspiro[4.4]nonane-1,6-dione or the corresponding substituted derivative in 96–100% yield.

**3,8-dibenzylidene-2,7-dioxaspiro[4.4]nonane-1,6-dione (5a):**^1^H NMR (DMSO): δ 7.52 (s, 2H), 7.38 (d, *J* = 6.2 Hz, 2H), 7.25 (s, 1H), 5.87 (s, 1H), 3.63 (d, *J* = 16.3 Hz, 1H), 3.37 (d, *J* = 22.0 Hz, 2H). ^13^C NMR (DMSO): δ 171.8, 146.0, 133.6, 128.6, 128.1, 126.9, 104.5, 36.4. HRMS (ESI Orbitrap) *m*/*z* 333.11245 [M + H]^+^ (calcd for C_21_H_17_O_4_^+^, 333.11214), 355.09397 [M + Na]^+^ (calcd for C_21_H_16_O_4_Na^+^, 355.09408).

**3,8-dimethylene-2,7-dioxaspiro[4.4]nonane-1,6-dione (5b):** white solid; mp: 60–61 °C. ^1^H NMR (DMSO): δ 4.82 (m, 2H), 4.54 (br s, 2H), 3.41 (d, *J* = 17.3 Hz, 2H), 3.14 (d, *J* = 17.3 Hz, 2H); ^13^C NMR (DMSO): δ 168.6, 131.7, 128.2, 62.1, 56.8, 36.1. HRMS (ESI Orbitrap) *m*/*z* 181.04937 [M + H]^+^ (calcd for C_9_H_9_O_4_^+^, 181.04954), 203.03167 [M + Na]^+^ (calcd for C_9_H_8_O_4_Na^+^, 203.03148).

**3,8-bis(4-methoxybenzylidene)-2,7-dioxaspiro[4.4]nonane-1,6-dione (5c):** white solid; mp: 50–51 °C. ^1^H NMR (DMSO): δ 7.26 (t, *J* = 8.0 Hz, 2H), 7.10–7.05 (m, 4H), 6.80 (dd, *J*_1,2_ = 6.0 Hz, *J*_2,3_ = 2.0 Hz, 2H), 5.81 (s, 2H), 5.73 (s, 1H), 3.73 (s, 6H), 3.58 (d, *J* = 17.6 Hz, 2H), 3.32 (d, *J* = 17.6 Hz, 2H); ^13^C NMR (DMSO): δ 172.2, 159.8, 146.6, 135.3, 130.1, 121.1, 114.2, 112.7, 104.9, 55.5, 50.6, 36.9. HRMS (ESI Orbitrap) *m*/*z* 393.13553 [M + H]^+^ (calcd for C_23_H_21_O_6_^+^, 393.13326), 415.11464 [M + Na]^+^ (calcd for C_23_H_20_NaO_6_^+^, 415.11521).

**3-benzylidene-8-(3-methoxybenzylidene)-2,7-dioxaspiro[4.4]nonane-1,6-dione (5d):** white solid; mp: 40–41 °C. ^1^H NMR (DMSO): δ 7.53 (d, *J* = 7.2 Hz, 2H), 7.39–7.25 (m, 4H), 7.13–7.08 (m, 2H), 6.84 (d, *J* = 7.2 Hz, 1H), 5.87–5.85 (m, 2H), 3.76 (s, 3H), 3.62 (d, *J* = 17.2 Hz, 2H), 3.37 (d, *J* = 17.2 Hz, 2H); ^13^C NMR (DMSO): δ 172.2, 159.8, 146.6, 146.4, 135.3, 134.0, 130.1, 129.1, 128.6, 127.3, 121.1, 114.3, 112.7, 105.0, 104.9, 55.5, 50.6, 36.9. HRMS (ESI Orbitrap) *m*/*z* 363.12143 [M + H]^+^ (calcd for C_22_H_19_O_5_^+^, 363.12270), 385.10529 [M + Na]^+^ (calcd for C_22_H_18_NaO_5_^+^, 385.10464).

**3-(4-acetylbenzylidene)-8-benzylidene-2,7-dioxaspiro[4.4]nonane-1,6-dione (5e):** white solid; mp: 45–46 °C. ^1^H NMR (DMSO): δ 7.97 (d, *J* = 8.0 Hz, 2H), 7.65 (d, *J* = 8.0 Hz, 2H), 7.52 (d, *J* = 7.2 Hz, 2H), 7.38 (t, *J* = 7.6 Hz, 2H), 7.24 (t, *J* = 7.6 Hz, 1H), 5.98 (s, 1H), 5.88 (s, 1H), 3.69–3.61 (m, 2H), 3.43–3.35 (m, 4H); ^13^C NMR (DMSO): δ 197.7, 172.2, 172.1, 148.9, 146.4, 138.8, 135.3, 134.0, 129.1, 129.1, 128.6, 127.3, 105.0, 104.2, 50.6, 37.1, 36.9, 27.1. HRMS (ESI Orbitrap) *m*/*z* 375.12211 [M + H]^+^ (calcd for C_23_H_19_O_5_^+^, 375.12270), 397.10425 [M + Na]^+^ (calcd for C_23_H_18_NaO_5_^+^, 397.10464).

**3,8-bis(4-methylbenzylidene)-2,7-dioxaspiro[4.4]nonane-1,6-dione (5f):** white solid; mp: 55–56 °C. ^1^H NMR (DMSO): δ 7.42 (d, *J* = 7.8 Hz, 4H), 7.19 (d, *J* = 7.2 Hz, 4H), 5.81 (s, 2H), 3.60 (d, *J* = 16.8 Hz, 2H), 3.36 (d, *J* = 16.8 Hz, 2H), 2.30 (s, 6H); ^13^C NMR (DMSO): δ 172.3, 145.6, 136.6, 131.2, 129.6, 128.5, 104.9, 50.7, 36.9, 21.2. HRMS (ESI Orbitrap) *m*/*z* 361.14517 [M + H]^+^ (calcd for C_23_H_21_O_4_^+^, 361.14344), 383.12479 [M + Na]^+^ (calcd for C_23_H_20_NaO_4_^+^, 383.12538).

**3,8-bis(naphthalen-1-ylmethylene)-2,7-dioxaspiro[4.4]nonane-1,6-dione (5g):** white solid; mp: 45–46 °C. ^1^H NMR (DMSO): δ 8.20 (d, *J* = 7.6 Hz, 2H), 7.96 (d, *J* = 7.4 Hz, 1H), 7.87 (d, *J* = 8.2 Hz, 1H), 7.79 (d, *J* = 7.1 Hz, 1H), 7.57 (m, 6H), 6.61 (s, 1H), 3.80 (d, *J* = 17.2 Hz, 1H), 3.55 (d, *J* = 17.2 Hz, 1H); ^13^C NMR (DMSO): δ 172.4, 147.9, 133.8, 131.1, 130.4, 128.9 127.8, 127.1, 126.7, 126.4, 126.0, 124.6, 101.7, 51.3, 36.9. HRMS (ESI Orbitrap) *m*/*z* 433.14383 [M + H]^+^ (calcd for C_29_H_21_O_4_^+^, 433.14344), 455.12643 [M + Na]^+^ (calcd for C_29_H_20_NaO_4_^+^, 455.12538).

**3,8-bis(3-methoxybenzylidene)-2,7-dioxaspiro[4.4]nonane-1,6-dione (5h):** white solid; mp: 65–66 °C. ^1^H NMR (DMSO): δ 7.30 (t, *J* = 8.0 Hz, 2H), 7.13 (d, *J* = 7.7 Hz, 2H), 7.09 (s, 2H), 6.88–6.82 (m, 2H), 5.85 (s, 2H), 3.77 (s, 3H) 3.62 (d, *J* = 17.4 Hz, 2H), 3.37 (d, *J* = 17.4 Hz, 2H); ^13^C NMR (DMSO): δ 172.2, 159.8, 146.6, 135.3, 130.1, 121.1, 114.3, 112.8, 104.9, 55.5, 50.6, 36.9. HRMS (ESI Orbitrap) *m*/*z* 393,13415 [M + H]^+^ (calcd for C_23_H_21_O_6_^+^, 393,13326), 415.11583 [M + Na]^+^ (calcd for C_23_H_20_NaO_6_^+^, 415.11521).

**3,8-bis(4-bromo-3-fluorobenzylidene)-2,7-dioxaspiro[4.4]nonane-1,6-dione (5i):** white solid; mp: 65–66 °C. ^1^H NMR (DMSO): δ 7.72 (t, *J* = 8.0 Hz, 2H), 7.46 (d, *J* = 10.4 Hz, 2H), 7.33 (d, *J* = 8.4 Hz, 2H), 5.92 (s, 2H), 3.64 (d, *J* = 17.6 Hz, 2H), 3.38 (d, *J* = 17.6 Hz, 2H); ^13^C NMR (DMSO): δ 171.8, 159.9, 157.5, 148.4, 136.0, 135.9, 134.1, 126.2, 116.2, 116.0, 106.4, 106.2, 103.1, 50.5, 36.9. HRMS (ESI Orbitrap) *m*/*z* 524.91356 [M + H]^+^ (calcd for C_21_H_13_Br_2_F_2_O_4_^+^, 524.91432), 546.89517 [M + Na]^+^ (calcd for C_21_H_12_Br_2_F_2_NaO_4_^+^, 546.89626).

**3,8-bis(4-chlorobenzylidene)-2,7-dioxaspiro[4.4]nonane-1,6-dione (5j):** white solid; mp: 65–66 °C. ^1^H NMR (DMSO): δ 7.51 (d, *J* = 7.6 Hz, 4H), 7.41 (d, *J* = 7.6 Hz, 4H), 5.86 (m, 2H), 3.59 (d, *J* = 17.2 Hz, 2H), 3.34 (d, *J* = 17.2 Hz, 2H); ^13^C NMR (DMSO): δ 172.0, 147.2, 133.0, 131.7, 130.2, 129.1, 103.8, 50.6, 36.9. HRMS (ESI Orbitrap) *m*/*z* 401.03474 [M + H]^+^ (calcd for C_21_H_15_Cl_2_O_4_^+^, 401.03419), 423.01581 [M + Na]^+^ (calcd for C_21_H_14_Cl_2_NaO_4_^+^, 423.01614).

**3,8-bis(4-bromobenzylidene)-2,7-dioxaspiro[4.4]nonane-1,6-dione (5k):** white solid; mp: 67–68 °C. ^1^H NMR (DMSO): δ 7.58 (d, *J* = 8.4 Hz, 4H), 7.48 (d, *J* = 8.4 Hz, 4H), 5.88 (s, 2H), 3.62 (d, *J* = 18.0 Hz, 2H), 3.35 (d, *J* = 18.0 Hz, 2H); ^13^C NMR (DMSO): δ 172.0, 147.3, 133.3, 132.0, 130.5, 120.2, 103.9, 50.6, 36.9. HRMS (ESI Orbitrap) *m*/*z* 488.93451 [M + H]^+^ (calcd for C_21_H_15_Br_2_O_4_^+^, 488.93316), 510.91493 [M + Na]^+^ (calcd for C_21_H_14_Br_2_NaO_4_^+^, 510.91511).

**3,8-bis(2-methyl-5-nitrobenzylidene)-2,7-dioxaspiro[4.4]nonane-1,6-dione (5l):** white solid; mp: 57–58 °C. ^1^H NMR (DMSO): δ 8.46 (d, *J* = 2.4 Hz, 2H), 8.03 (dd, *J_1,2_* = 6.0 Hz, *J*_2,3_ = 2.4 Hz, 2H), 7.53 (d, *J* = 8.8 Hz, 2H), 6.09 (s,2H), 3.75 (d, *J* = 18.0 Hz, 2H), 3.46 (d, *J* = 18.0 Hz, 2H), 2.45 (s, 6H); ^13^C NMR (DMSO): δ 171.9, 149.0, 146.4, 144.2, 134.1, 131.8, 123.2, 122.0, 100.9, 50.9, 37.0, 20.4. HRMS (ESI Orbitrap) *m*/*z* 451.11426 [M + H]^+^ (calcd for C_23_H_19_N_2_O_8_^+^, 451.11359), 473.09627 [M + Na]^+^ (calcd for C_23_H_18_N_2_NaO_8_^+^, 473.09554).

**3-(4-chlorobenzylidene)-8-(4-methoxybenzylidene)-2,7-dioxaspiro[4.4]nonane-1,6-dione (5m):** white solid; mp: 55–56 °C. ^1^H NMR (DMSO): δ 7.54 (d, *J* = 8.4 Hz, 2H), 7.46 (t, *J* = 7.2 Hz, 4H), 6.95 (d, *J* = 8.8 Hz, 2H), 5.89 (s,1H), 5.80 (s, 1H), 3.76 (s, 3H), 3.60 (d, *J* = 18.0 Hz, 2H), 3.46 (d, *J* = 18.0 Hz, 2H); ^13^C NMR (DMSO): δ 171.7, 171.6, 158.1, 146.7, 144.0, 132.5, 131.1, 129.7, 129.4, 128.6, 126.1, 125.7, 114.1, 114.0, 104.2, 103.4, 103.3, 97.3, 55.1, 50.2, 36.5, 36.3. HRMS (ESI Orbitrap) *m*/*z* 397.08365 [M + H]^+^ (calcd C_22_H_18_ClO_5_^+^, 397.08373), 419.06529 [M + Na]^+^ (calcd for C_22_H_17_ClNaO_5_^+^, 419.06567).

**3,8-bis(2-methoxybenzylidene)-2,7-dioxaspiro[4.4]nonane-1,6-dione (5n):** white solid; mp: 61–62 °C. ^1^H NMR (DMSO): δ 7.69 (dd, *J* = 7.8, 1.4 Hz, 2H), 7.29–7.22 (m, 2H), 7.03 (d, *J* = 8.3 Hz, 2H), 6.99 (t, *J* = 7.8 Hz, 2H), 6.04 (s, 2H), 3.82 (s, 6H), 3.67 (dd, *J* = 17.3, 1.6 Hz, 2H), 3.38 (dd, *J* = 17.3, 1.6 Hz, 2H). ^13^C NMR (DMSO): δ 172.3, 156.0, 146.3, 129.4, 128.8, 122.2, 120.9, 111.5, 98.9, 56.0, 50.8, 37.1. HRMS (ESI Orbitrap) *m*/*z* 393.13428 [M + H]^+^ (calcd for C_23_H_21_O_6_^+^, 393.13326), 415.11581 [M + Na]^+^ (calcd for C_23_H_20_NaO_6_^+^, 415.11521).

## 4. Conclusions

The activation of alkynes by gold(I) complexes is a versatile tool for the selective functionalization of C-C triple bond with several nucleophiles, including heteroatoms. The intramolecular version of this reaction leads to cyclic or heterocyclic intermediates, that are useful for the synthesis of high value pharmaceutically relevant compounds. We demonstrated that functionalized symmetric or unsymmetric biarylacetylenic malonic acids may be efficiently cyclized under extremely mild conditions at room temperature in presence of JohnPhosAu(MeCN)SbF_6_ catalyst, and without any additives. The corresponding γ-arylmethylene-spirobislactones were isolated in excellent yields (96–100%). This process constitutes an easy and efficient access to highly valuable building blocks of natural products or biologically active compounds. The high reactivity of gold(I) catalyst towards the activation of C-C triple bond, associated with very mild reactions conditions would allow further synthesis of lactones.

## Figures and Tables

**Table 1:**
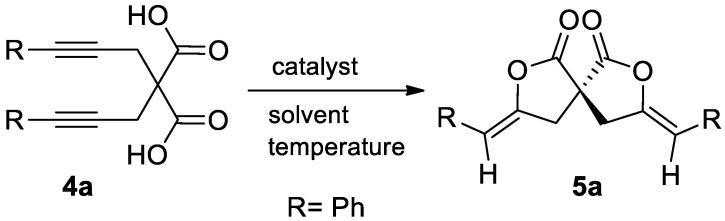
Screening for the best catalyst and reaction conditions for the spirodilactonization to γ-arylmethylene-spirobislactones **5**: cyclization of 2,2-bis(3-phenylprop-2-yn-1-yl)malonic acid **4a** to 3,8-benzylidene-2,7-dioxaspiro[4.4]nonane-1,6-diones **5a** ^a,b^.

Entry	Catalyst	Base	Solvent	T (°C)	t (h)	Yield 5a (%)
**1**	PdCl_2_(CH_3_CN)_2_	K_2_CO_3_ ^c^	MeCN	80	24	-
**2**	NaAuCl_4_	-	EtOH	100	12	-
**3**	JohnPhosAu (MeCN)SbF_6_	-	DCM	80	1	90
**4**	JohnPhosAu (MeCN)SbF_6_ ^d^	-	DCM	40	1	99
**5**	JohnPhosAu (MeCN)SbF_6_ ^d^	-	DCM	rt	1	100

^a^ Reactions were carried out on a 0.5 mmol scale in 2.0 mL of solvent using 0.05 equiv. of catalyst. ^b^ Yields are given for isolated compounds. ^c^ 2.0 equiv. of base were used. ^d^ 0.02 equiv of catalyst were used.

**Table 2:**
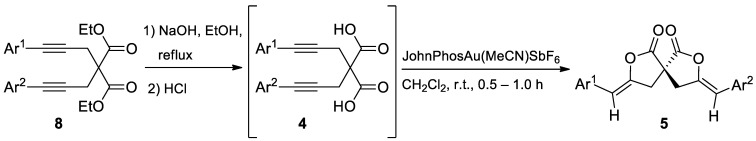
Substrate scope for the gold(I)-catalyzed spirodilactonization to γ-arylidenespirobislactones **5** ^a,b^.

Entry	Ar^1^	Ar^2^	t (h)	Yield of 5 (%)
**1**	Ph	Ph	1	**5a** 100
**2**	H	H	1	**5b** 100
**3**	4-OCH_3_-C_6_H_4_-	4-OCH_3_-C_6_H_4_-	0.5	**5c** 96
**4**	3-OCH_3_-C_6_H_4_-	Ph-	1	**5d** 98
**5**	Ph-	4-COCH_3_-C_6_H_4_-	0.5	**5e** 99
**6**	4-CH_3_-C_6_H_4_	4-CH_3_-C_6_H_4_-	1	**5f** 98
**7**	1-naphtyl	1-naphtyl	1	**5g** 100
**8**	3-OCH_3_-C_6_H_4_-	3-OCH_3_-C_6_H_4_-	1	**5h** 97
**9**	4-Br-3-F-C_6_H_3_-	4-Br-3-F-C_6_H_2_-	0.5	**5i** 100
**10**	4-Cl-C_6_H_4_-	4-Cl-C_6_H_4_-	1	**5j** 100
**11**	4-Br-C_6_H_4_-	4-Br-C_6_H_4_-	1	**5k** 99
**12**	2-CH_3_,5-NO_2_-C_6_H_3_-	2-CH_3_,5-NO_2_-C_6_H_3_-	1	**5l** 98
**13**	4-Cl-C_6_H_4_-	4-OCH_3_-C_6_H_4_-	1	**5m** 97
**14**	2-OCH_3_-C_6_H_4_-	2-OCH_3_-C_6_H_4_-	1	**5n** 98

^a^ Reactions were carried out on a 0.5 mmol scale in 2.0 mL of CH_2_Cl_2_ using 0.02 equiv. of catalyst at rt, under air. ^b^ Yields are given for isolated compounds.

## Data Availability

Data is contained within the article or supplementary material.

## Supplementary Materials

The following supporting information can be downloaded at: https://www.mdpi.com/article/10.3390/molecules28010300/s1, Figure S1: ^1^H NMR of 3,8-dibenzylidene-2,7-dioxaspiro[4.4]nonane-1,6-dione (**5a**); Figure S2: ^13^C NMR of 3,8-dibenzylidene-2,7-dioxaspiro[4.4]nonane-1,6-dione (**5a**); Figure S3: ^1^H NMR of 3,8-dimethylene-2,7-dioxaspiro[4.4]nonane-1,6-dione (**5b**); Figure S4: ^13^C NMR of 3,8-dimethylene-2,7-dioxaspiro[4.4]nonane-1,6-dione (**5b**); Figure S5: ^1^H NMR of 3,8-bis(4-methoxybenzylidene)-2,7-dioxaspiro[4.4]nonane-1,6-dione (**5c**); Figure S6: ^13^C NMR of 3,8-bis(4-methoxybenzylidene)-2,7-dioxaspiro[4.4]nonane-1,6-dione (**5c**); Figure S7: ^1^H NMR of 3-benzylidene-8-(3-methoxybenzylidene)-2,7-dioxaspiro[4.4]nonane-1,6-dione (**5d**); Figure S8: ^13^C NMR of 3-benzylidene-8-(3-methoxybenzylidene)-2,7-dioxaspiro[4.4]nonane-1,6-dione (**5d**); Figure S9: ^1^H NMR of 3-(4-acetylbenzylidene)-8-benzylidene-2,7-dioxaspiro[4.4]nonane-1,6-dione (**5e**); Figure S10: ^13^C NMR of 3-(4-acetylbenzylidene)-8-benzylidene-2,7-dioxaspiro[4.4]nonane-1,6-dione (**5e**); Figure S11: ^1^H NMR of 3,8-bis(4-methylbenzylidene)-2,7-dioxaspiro[4.4]nonane-1,6-dione (**5f**); Figure S12: ^13^C NMR of 3,8-bis(4-methylbenzylidene)-2,7-dioxaspiro[4.4]nonane-1,6-dione (**5f**); Figure S13: ^1^H NMR of 3,8-bis(naphthalen-1-ylmethylene)-2,7-dioxaspiro[4.4]nonane-1,6-dione (**5g**); Figure S14: ^1^H NMR of 3,8-bis(naphthalen-1-ylmethylene)-2,7-dioxaspiro[4.4]nonane-1,6-dione (**5g**); Figure S15: ^1^H NMR of 3,8-bis(3-methoxybenzylidene)-2,7-dioxaspiro[4.4]nonane-1,6-dione (**5h**); Figure S16: ^13^C NMR of 3,8-bis(3-methoxybenzylidene)-2,7-dioxaspiro[4.4]nonane-1,6-dione (**5h**); Figure S17: ^1^H NMR of 3,8-bis(4-bromo-3-fluorobenzylidene)-2,7-dioxaspiro[4.4]nonane-1,6-dione (**5i**); Figure S18: ^13^C NMR of 3,8-bis(4-bromo-3-fluorobenzylidene)-2,7-dioxaspiro[4.4]nonane-1,6-dione (**5i**); Figure S19: ^1^H NMR of 3,8-bis(4-chlorobenzylidene)-2,7-dioxaspiro[4.4]nonane-1,6-dione (**5j**); Figure S20: ^13^C NMR of 3,8-bis(4-chlorobenzylidene)-2,7-dioxaspiro[4.4]nonane-1,6-dione (**5j**); Figure S21: ^1^H NMR of 3,8-bis(4-bromobenzylidene)-2,7-dioxaspiro[4.4]nonane-1,6-dione (**5k**); Figure S22: ^13^C NMR of 3,8-bis(4-bromobenzylidene)-2,7-dioxaspiro[4.4]nonane-1,6-dione (**5k**); Figure S23: ^1^H NMR of 3,8-bis(2-methyl-5-nitrobenzylidene)-2,7-dioxaspiro[4.4]nonane-1,6-dione (**5l**); Figure S24. ^13^C NMR of 3,8-bis(2-methyl-5-nitrobenzylidene)-2,7-dioxaspiro[4.4]nonane-1,6-dione (**5l**); Figure S25: ^1^H NMR of 3-(4-chlorobenzylidene)-8-(4-methoxybenzylidene)-2,7-dioxaspiro[4.4]nonane-1,6-dione (**5m**); Figure S26: ^13^C NMR of 3-(4-chlorobenzylidene)-8-(4-methoxybenzylidene)-2,7-dioxaspiro[4.4]nonane-1,6-dione (**5m**); Figure S27: ^1^H NMR of 3,8-bis(2-methoxybenzylidene)-2,7-dioxaspiro[4.4]nonane-1,6-dione (**5n**); Figure S28: ^1^H NMR of 3,8-bis(2-methoxybenzylidene)-2,7-dioxaspiro[4.4]nonane-1,6-dione (**5n**).
