# Supplementary material for: Highly Efficient and Mild Gold (I) Catalyzed Synthesis of 3,8-Diarylidene-2,7-dioxaspiro[4.4]nonane-1,6-diones
Source: Molecules. 2022 Dec 30;28(1):300. doi: 10.3390/molecules28010300 (PMC9822471; doi:10.3390/molecules28010300)
Supplement: Supplementary file 1 [file molecules-28-00300-s001.zip › molecules-2127792-supplementary.pdf]

Highly efficient and mild Gold (I) catalyzed synthesis of 3,8-diarylidene-2,7-dioxaspiro[4.4]nonane-1,6-diones

Antonia Iazzetti[1], Dario Allevi[1], Andrea Calcaterra[2,*] , Giancarlo Fabrizi[2], Antonella Goggiamani[2], Giulia Mazzoccanti[2], Alessio Sferrazza[2,†], Rosanna Verdiglione[2], Valeria Vergine[2]

[1] Dipartimento di Scienze Biotecnologiche di Base Cliniche Intensivologiche e Perioperatorie, Università Cattolica del Sacro Cuore, Campus di Roma, Largo Francesco Vito 1, 00168 Rome, Italy

[2] Department of Chemistry and Technology of Drugs, Sapienza – University of Rome, P.le Aldo Moro 5, 00185 Rome, Italy

[†] Alessio Sferrazza is currently a research scientist at IRBM S.p.A., Medicinal Chemistry Department, Via Pontina km 30, n. 600, 00071 Pomezia (Rome), Italy

* Correspondence: Andrea Calcaterra, e-mail: andrea.calcaterra@uniroma1.it

Index:

		Pag.
**Figure S1.**	^{1}H NMR of 3,8-dibenzylidene-2,7-dioxaspiro[4.4]nonane-1,6-dione (**5a**)	S3
**Figure S2.**	^{13}C NMR of 3,8-dibenzylidene-2,7-dioxaspiro[4.4]nonane-1,6-dione (**5a**)	S3
**Figure S3.**	^{1}H NMR of 3,8-dimethylene-2,7-dioxaspiro[4.4]nonane-1,6-dione (**5b**)	S4
**Figure S4.**	^{13}C NMR of 3,8-dimethylene-2,7-dioxaspiro[4.4]nonane-1,6-dione (**5b**)	S4
**Figure S5.**	^{1}H NMR of 3,8-bis(4-methoxybenzylidene)-2,7-dioxaspiro[4.4]nonane-1,6-dione (**5c**)	S5
**Figure S6.**	^{13}C NMR of 3,8-bis(4-methoxybenzylidene)-2,7-dioxaspiro[4.4]nonane-1,6-dione (**5c**)	S5
**Figure S7.**	^{1}H NMR of 3-benzylidene-8-(3-methoxybenzylidene)-2,7-dioxaspiro[4.4]nonane-1,6-dione (**5d**)	S6
**Figure S8.**	^{13}C NMR of 3-benzylidene-8-(3-methoxybenzylidene)-2,7-dioxaspiro[4.4]nonane-1,6-dione (**5d**)	S6
**Figure S9.**	^{1}H NMR of 3-(4-acetylbenzylidene)-8-benzylidene-2,7-dioxaspiro[4.4]nonane-1,6-dione (**5e**)	S7
**Figure S10.**	^{13}C NMR of 3-(4-acetylbenzylidene)-8-benzylidene-2,7-dioxaspiro[4.4]nonane-1,6-dione (**5e**)	S7
**Figure S11.**	^{1}H NMR of 3,8-bis(4-methylbenzylidene)-2,7-dioxaspiro[4.4]nonane-1,6-dione (**5f**)	S8
**Figure S12.**	^{13}C NMR of 3,8-bis(4-methylbenzylidene)-2,7-dioxaspiro[4.4]nonane-1,6-dione (**5f**)	S8
**Figure S13.**	^{1}H NMR of 3,8-bis(naphthalen-1-ylmethylene)-2,7-dioxaspiro[4.4]nonane-1,6-dione (**5g**)	S9
**Figure S14.**	^{1}H NMR of 3,8-bis(naphthalen-1-ylmethylene)-2,7-dioxaspiro[4.4]nonane-1,6-dione (**5g**)	S9
**Figure S15.**	^{1}H NMR of 3,8-bis(3-methoxybenzylidene)-2,7-dioxaspiro[4.4]nonane-1,6-dione (**5h**)	S10
**Figure S16.**	^{13}C NMR of 3,8-bis(3-methoxybenzylidene)-2,7-dioxaspiro[4.4]nonane-1,6-dione (**5h**)	S10
**Figure S17.**	^{1}H NMR of 3,8-bis(4-bromo-3-fluorobenzylidene)-2,7-dioxaspiro[4.4]nonane-1,6-dione (**5i**)	S11

Figure S18. ^{13}C NMR of 3,8-bis(4-bromo-3-fluorobenzylidene)-2,7-dioxaspiro[4.4]nonane-1,6-dione (**5i**) S11

Figure S19. ^{1}H NMR of 3,8-bis(4-chlorobenzylidene)-2,7-dioxaspiro[4.4]nonane-1,6-dione (**5j**) S12

Figure S20. ^{13}C NMR of 3,8-bis(4-chlorobenzylidene)-2,7-dioxaspiro[4.4]nonane-1,6-dione (**5j**) S12

Figure S21. ^{1}H NMR of 3,8-bis(4-bromobenzylidene)-2,7-dioxaspiro[4.4]nonane-1,6-dione (**5k**) S13

Figure S22. ^{13}C NMR of 3,8-bis(4-bromobenzylidene)-2,7-dioxaspiro[4.4]nonane-1,6-dione (**5k**) S13

Figure S23. ^{1}H NMR of 3,8-bis(2-methyl-5-nitrobenzylidene)-2,7-dioxaspiro[4.4]nonane-1,6-dione (**5l**) S14

Figure S24. ^{13}C NMR of 3,8-bis(2-methyl-5-nitrobenzylidene)-2,7-dioxaspiro[4.4]nonane-1,6-dione (**5l**) S14

Figure S25. ^{1}H NMR of 3-(4-chlorobenzylidene)-8-(4-methoxybenzylidene)-2,7-dioxaspiro[4.4]nonane-1,6-dione (**5m**) S15

Figure S26. ^{13}C NMR of 3-(4-chlorobenzylidene)-8-(4-methoxybenzylidene)-2,7-dioxaspiro[4.4]nonane-1,6-dione (**5m**) S15

Figure S27. ^{1}H NMR of 3,8-bis(2-methoxybenzylidene)-2,7-dioxaspiro[4.4]nonane-1,6-dione (**5n**) S16

Figure S28. ^{1}H NMR of 3,8-bis(2-methoxybenzylidene)-2,7-dioxaspiro[4.4]nonane-1,6-dione (**5n**) S16

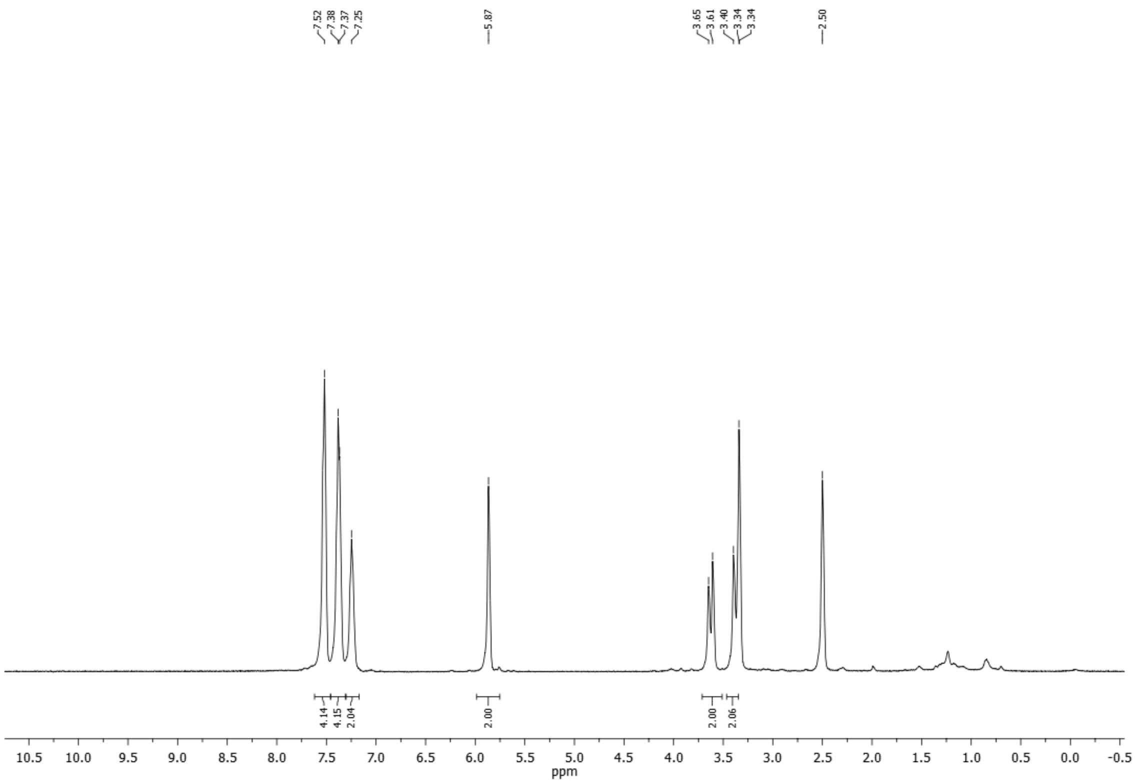

Figure S1. ^{1}H NMR of 3,8-dibenzylidene-2,7-dioxaspiro[4.4]nonane-1,6-dione (**5a**).

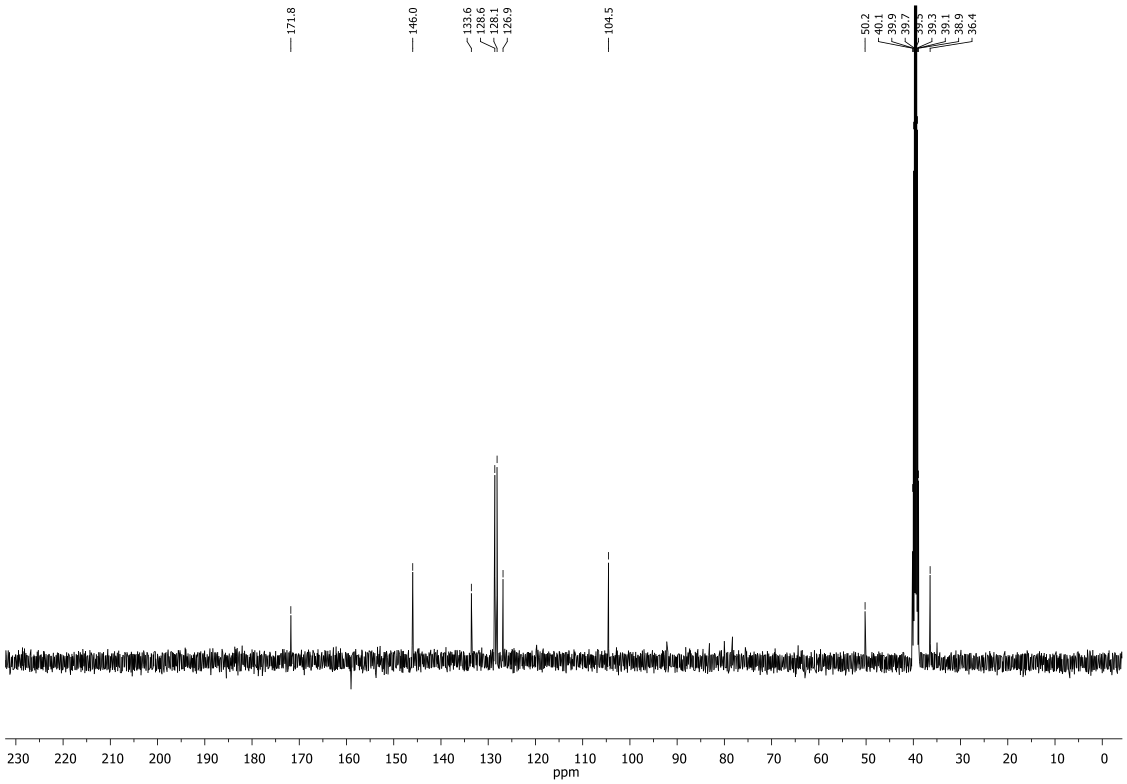

Figure S2. ^{13}C NMR of 3,8-dibenzylidene-2,7-dioxaspiro[4.4]nonane-1,6-dione (**5a**).

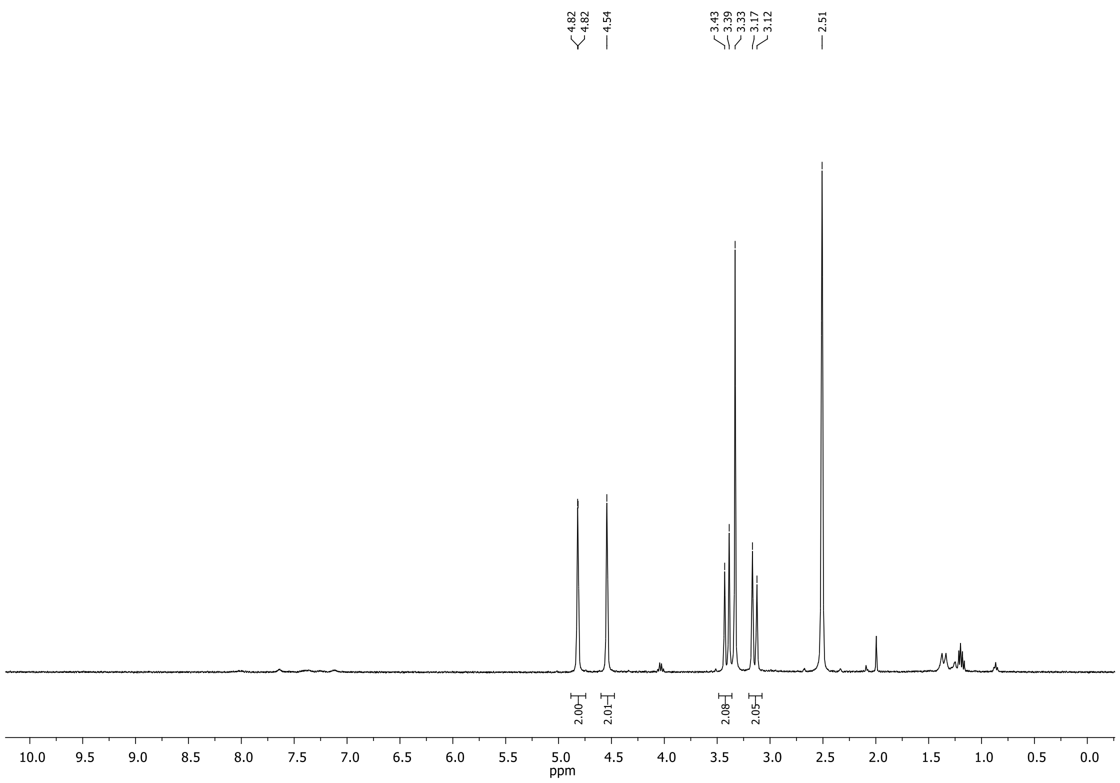

Figure S3. ^{1}H NMR of 3,8-dimethylene-2,7-dioxaspiro[4.4]nonane-1,6-dione (**5b**).

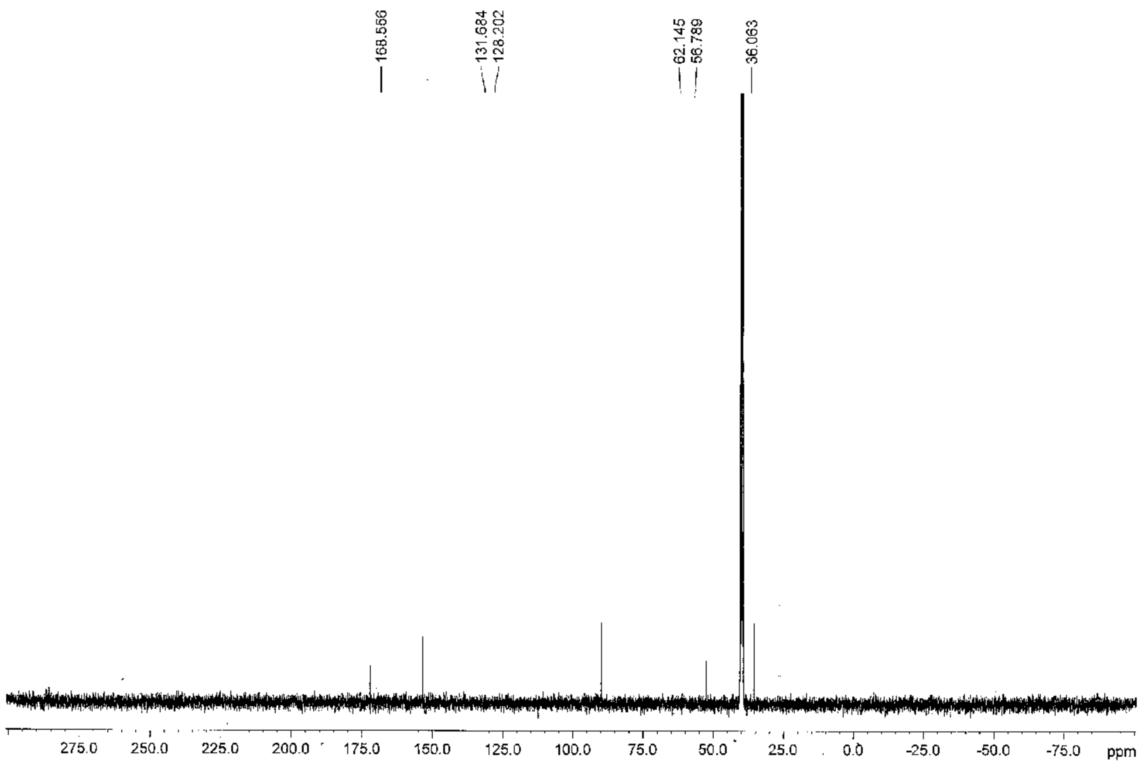

Figure S4. ^{13}C NMR of 3,8-dimethylene-2,7-dioxaspiro[4.4]nonane-1,6-dione (**5b**).

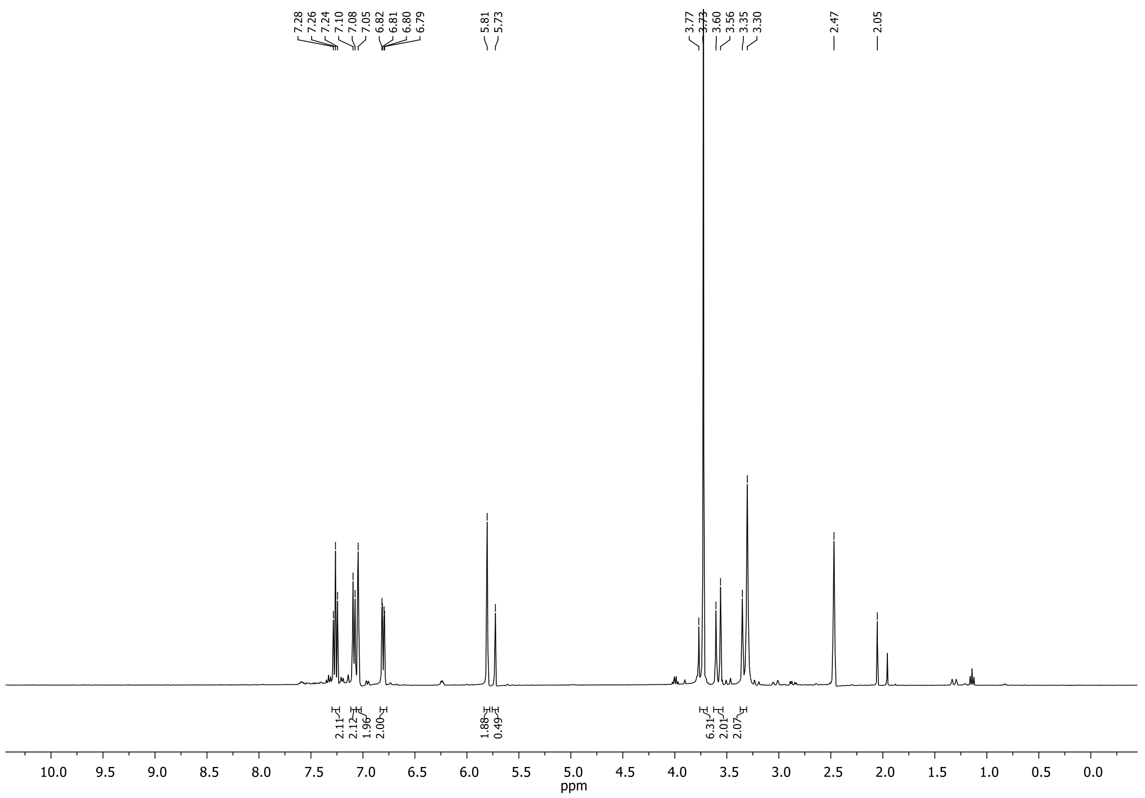

Figure S5. ^{1}H NMR of 3,8-bis(4-methoxybenzylidene)-2,7-dioxaspiro[4.4]nonane-1,6-dione (**5c**).

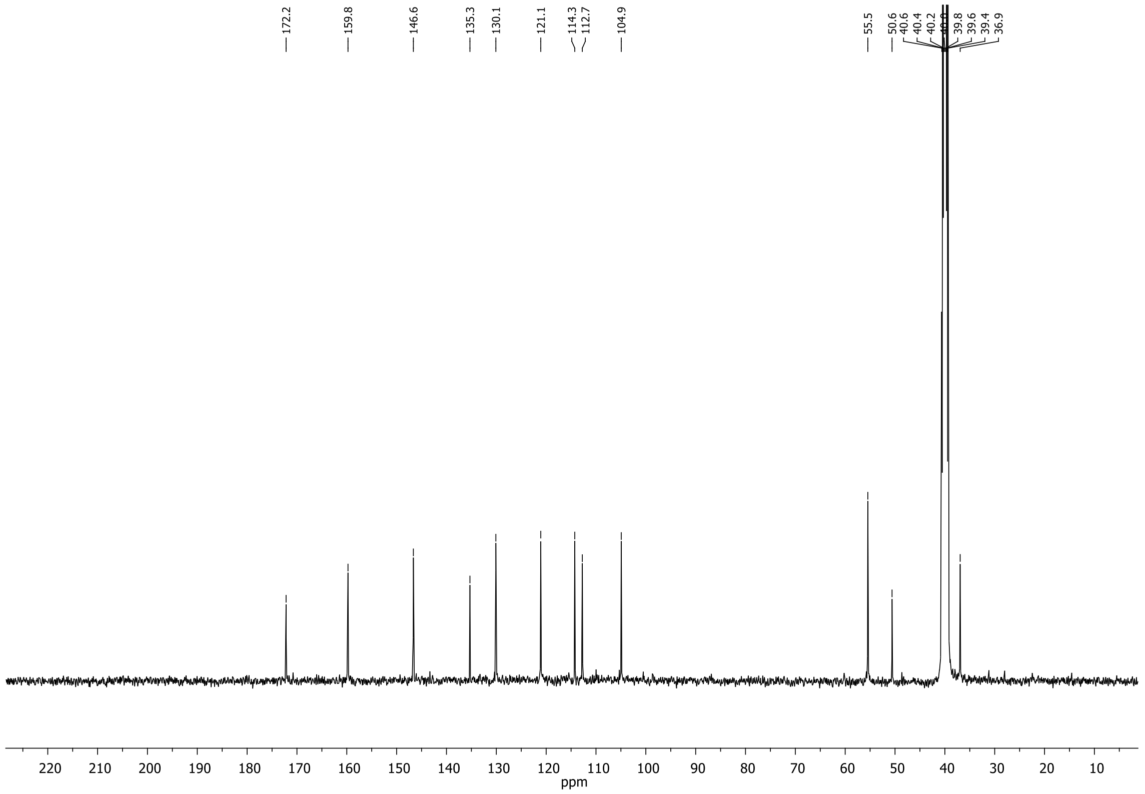

Figure S6. ^{13}C NMR of 3,8-bis(4-methoxybenzylidene)-2,7-dioxaspiro[4.4]nonane-1,6-dione (**5c**).

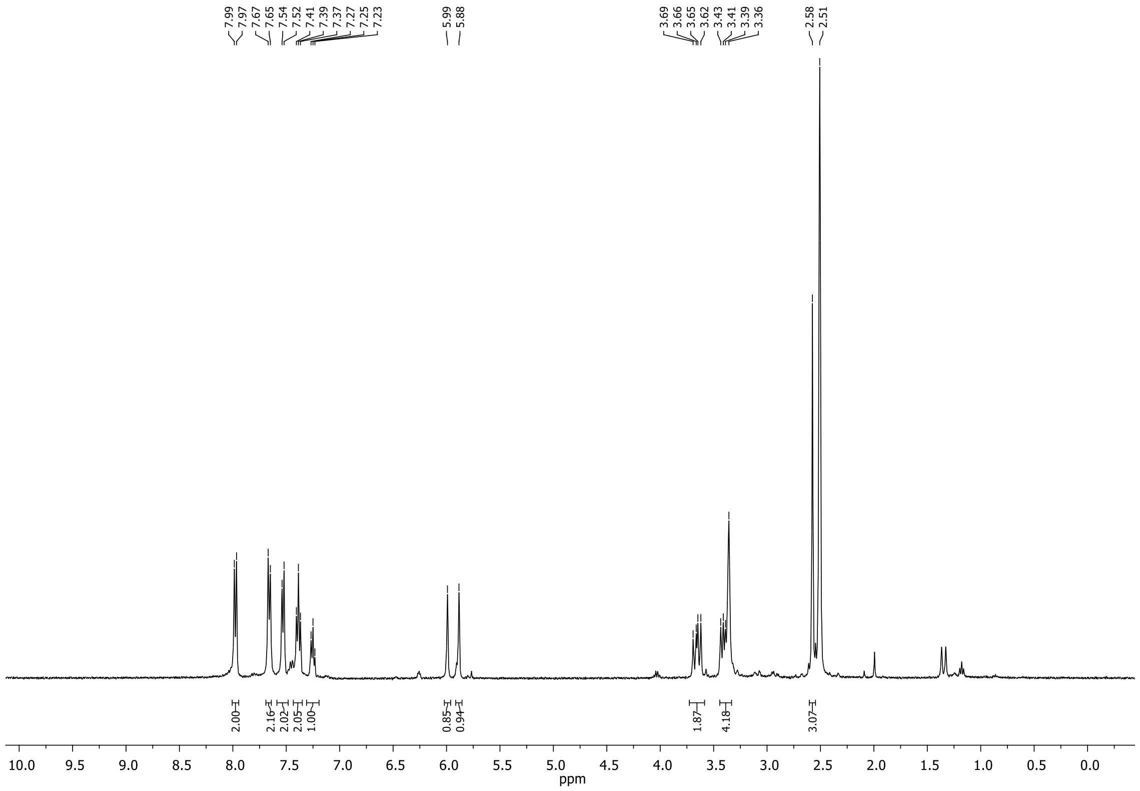

Figure S7. ^{1}H NMR of 3-benzylidene-8-(3-methoxybenzylidene)-2,7-dioxaspiro[4.4]nonane-1,6-dione (**5d**).

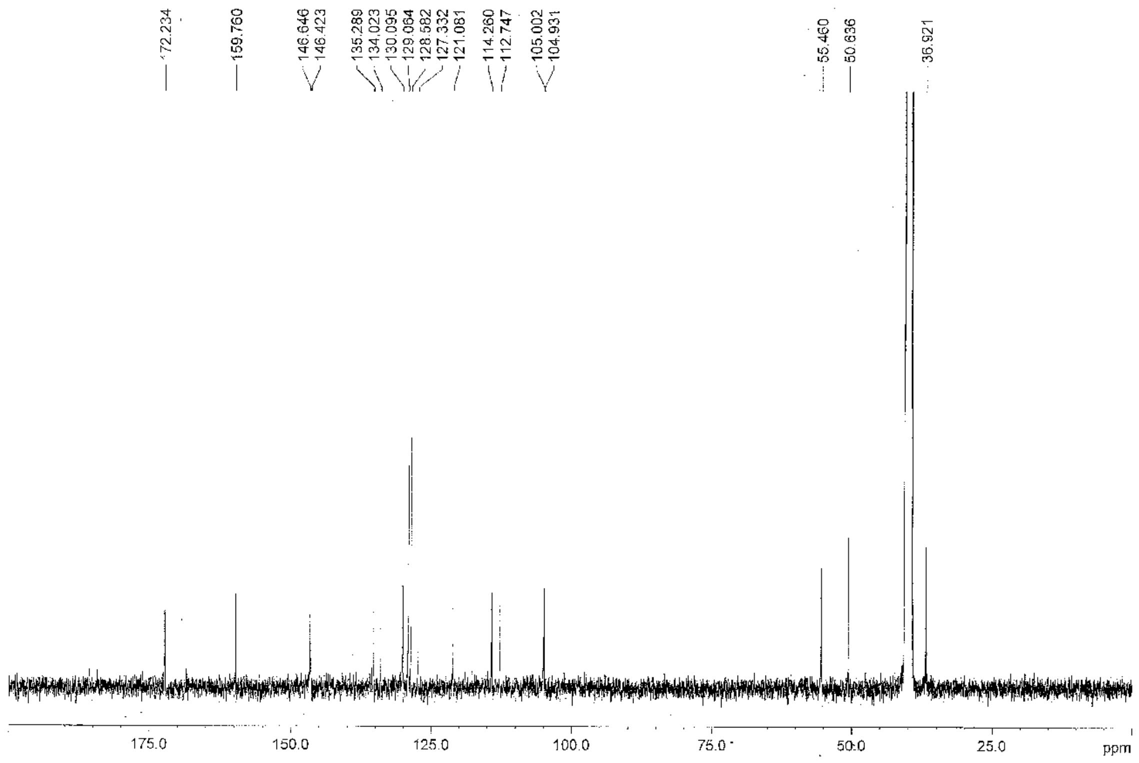

Figure S8. ^{13}C NMR of 3-benzylidene-8-(3-methoxybenzylidene)-2,7-dioxaspiro[4.4]nonane-1,6-dione (**5d**).

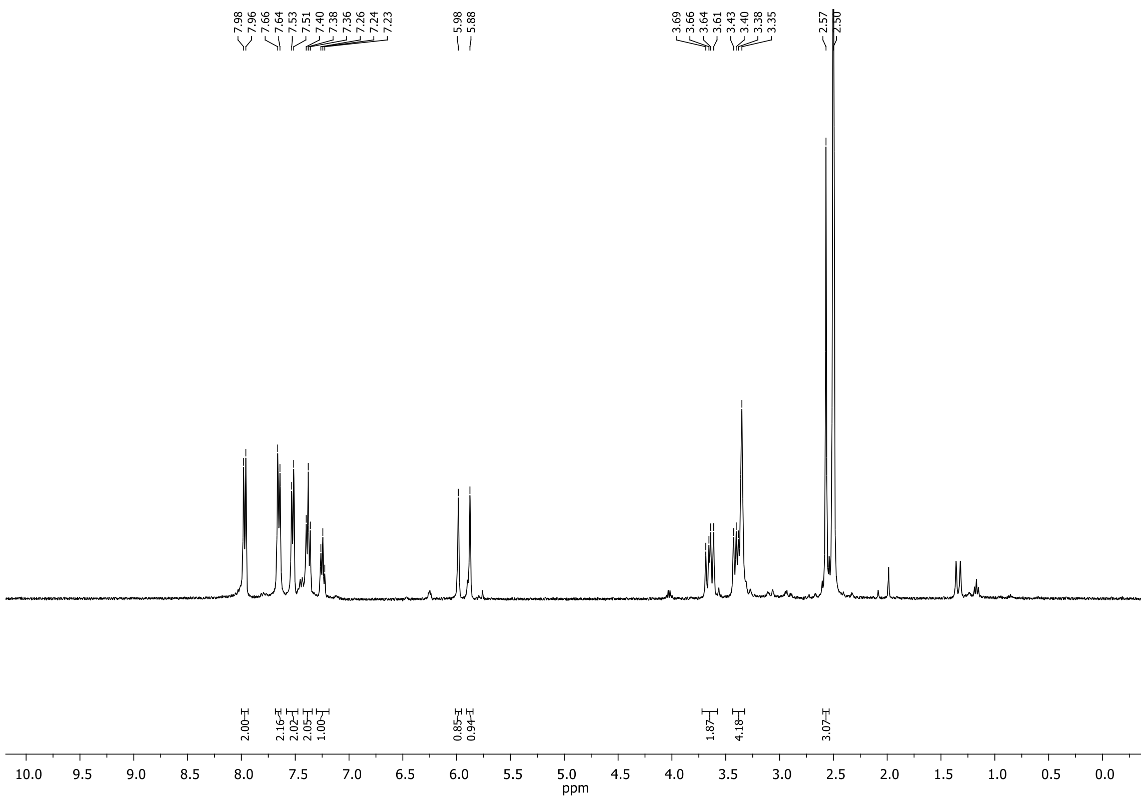

Figure S9. ^{1}H NMR of 3-(4-acetylbenzylidene)-8-benzylidene-2,7-dioxaspiro[4.4]nonane-1,6-dione (**5e**).

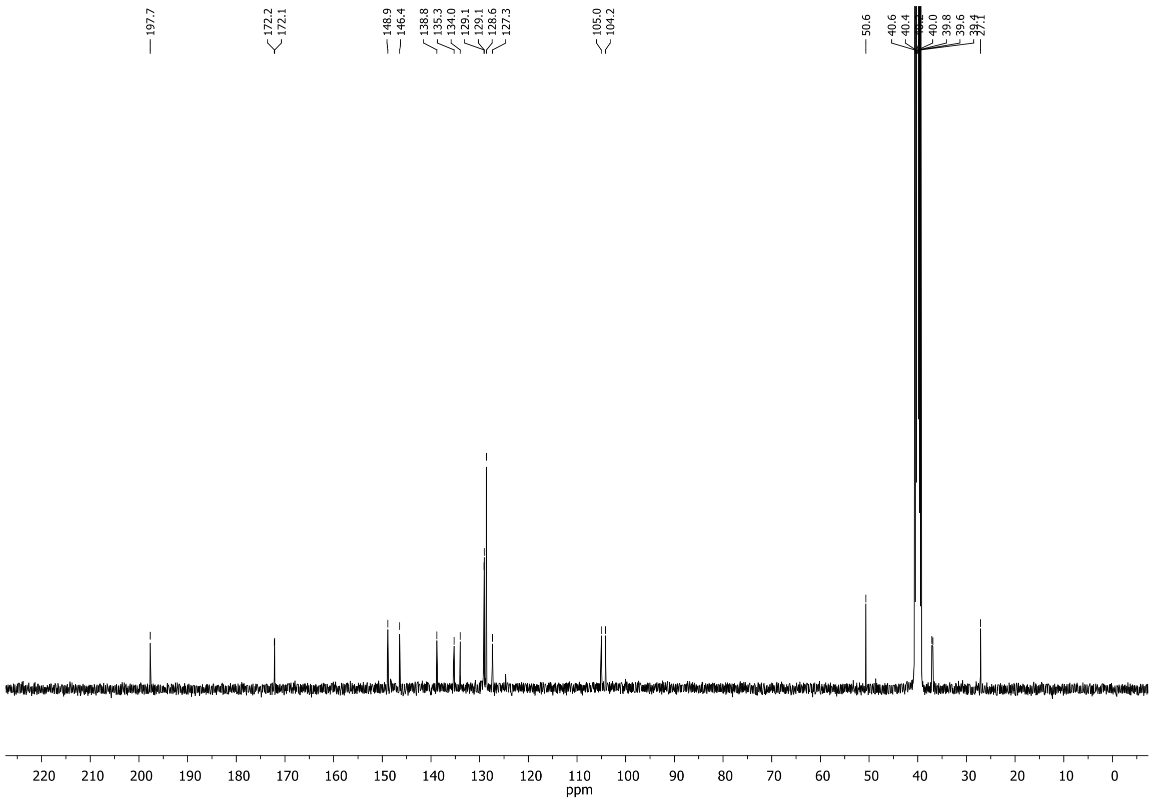

Figure S10. ^{13}C NMR of 3-(4-acetylbenzylidene)-8-benzylidene-2,7-dioxaspiro[4.4]nonane-1,6-dione (**5e**).

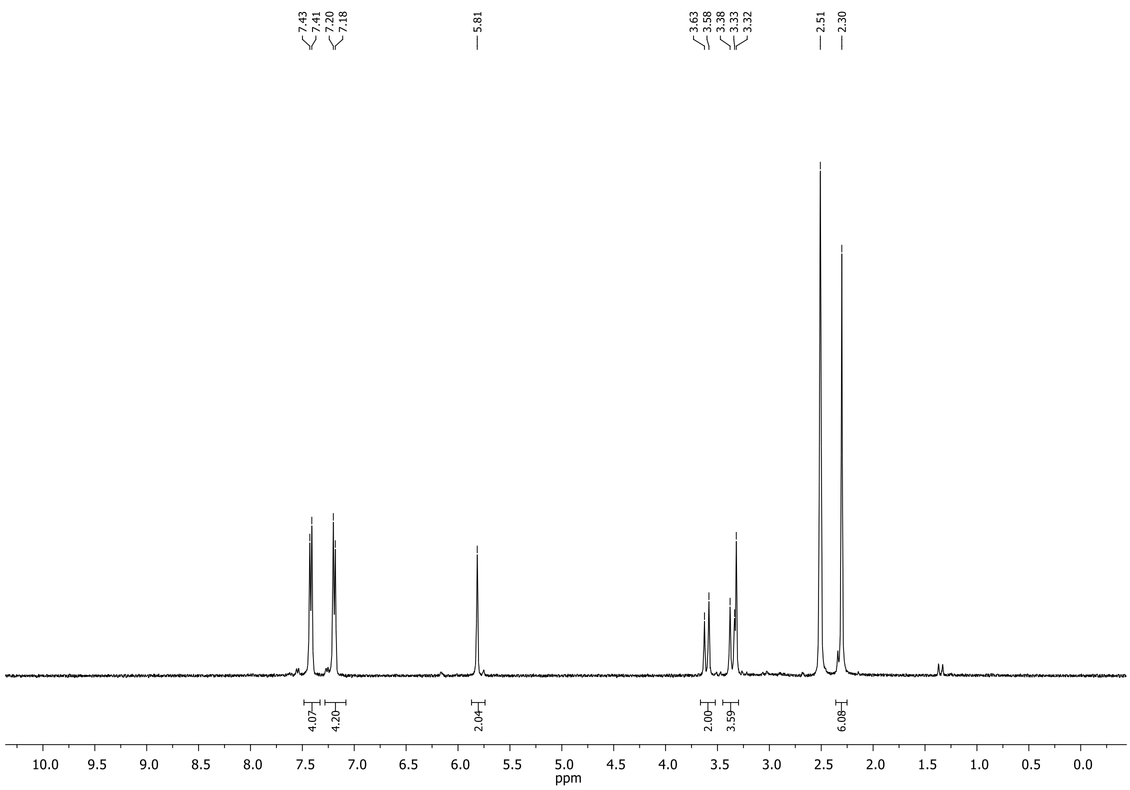

Figure S11. ^{1}H NMR of 3,8-bis(4-methylbenzylidene)-2,7-dioxaspiro[4.4]nonane-1,6-dione (**5f**).

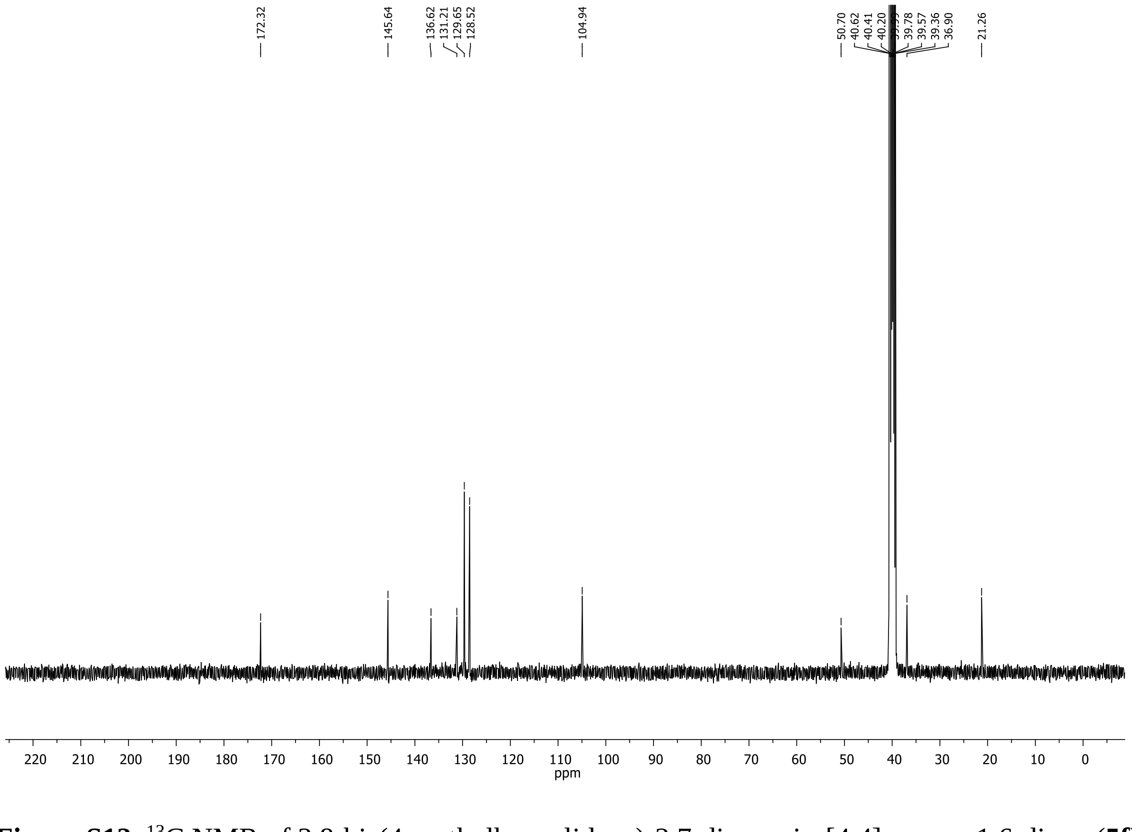

Figure S12. ^{13}C NMR of 3,8-bis(4-methylbenzylidene)-2,7-dioxaspiro[4.4]nonane-1,6-dione (**5f**).

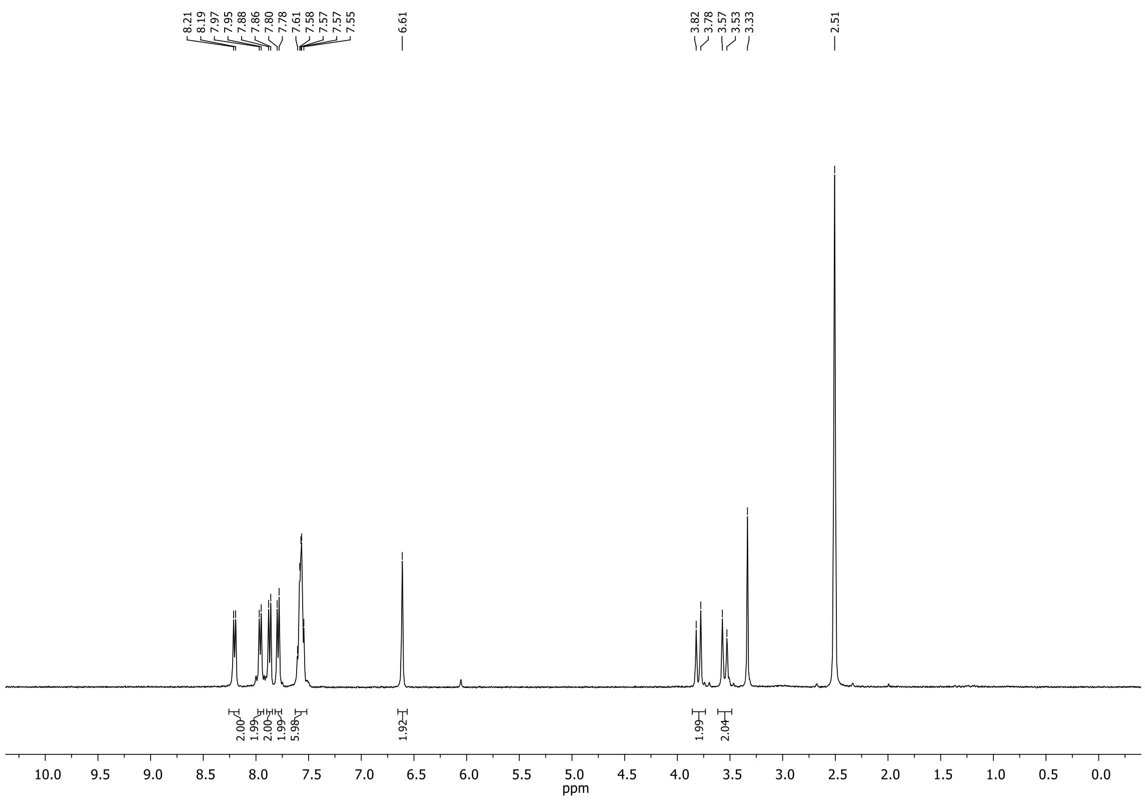

Figure S13. ^{1}H NMR of 3,8-bis(naphthalen-1-ylmethylene)-2,7-dioxaspiro[4.4]nonane-1,6-dione (**5g**).

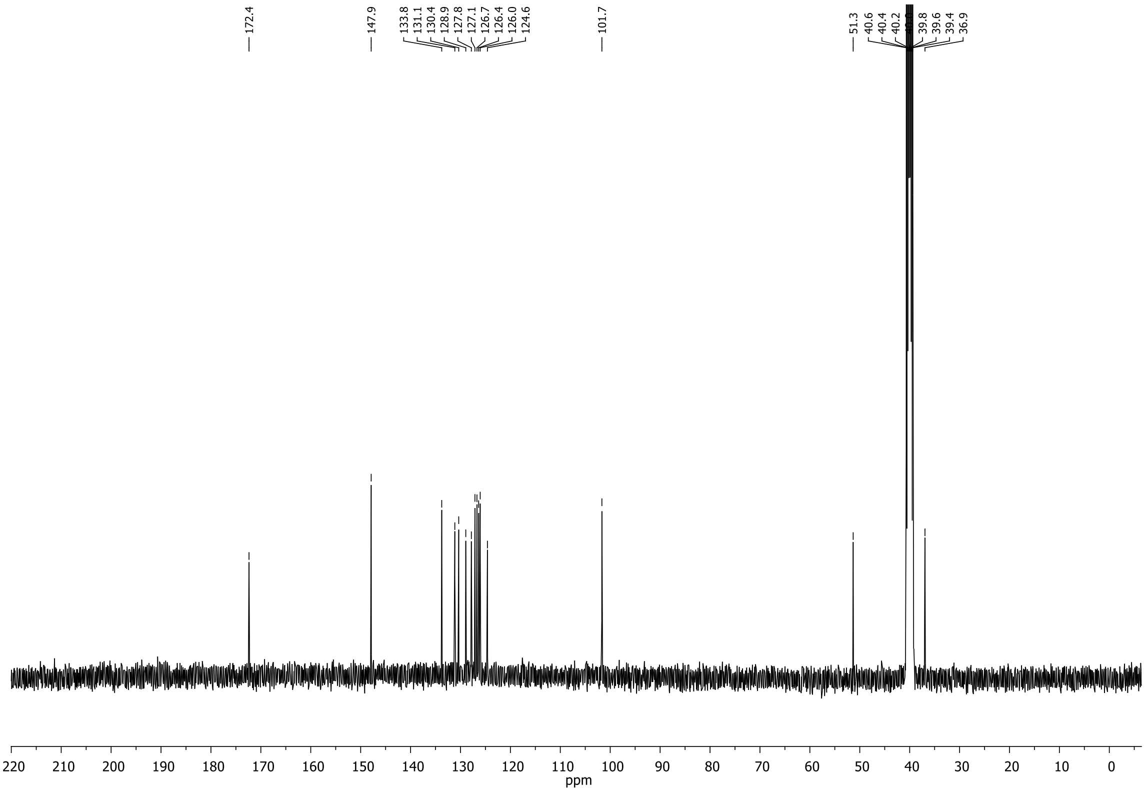

Figure S14. ^{13}C NMR of 3,8-bis(naphthalen-1-ylmethylene)-2,7-dioxaspiro[4.4]nonane-1,6-dione (**5g**).

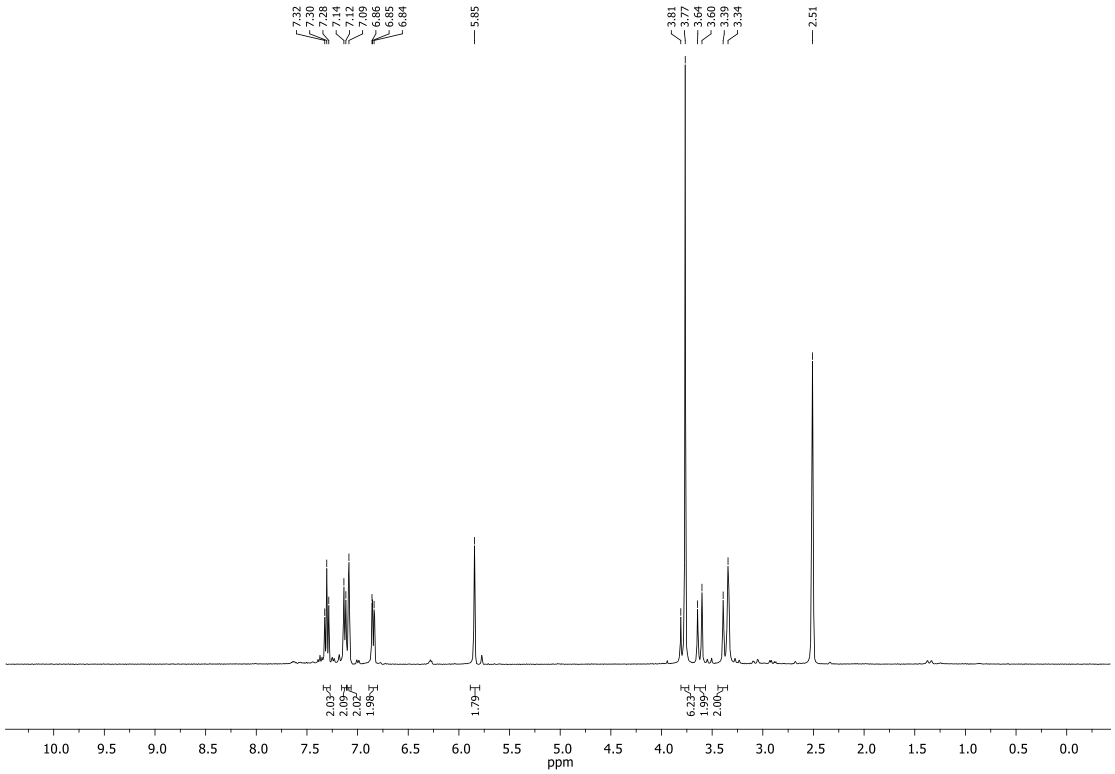

Figure S15. ^{1}H NMR of 3,8-bis(3-methoxybenzylidene)-2,7-dioxaspiro[4.4]nonane-1,6-dione (**5h**).

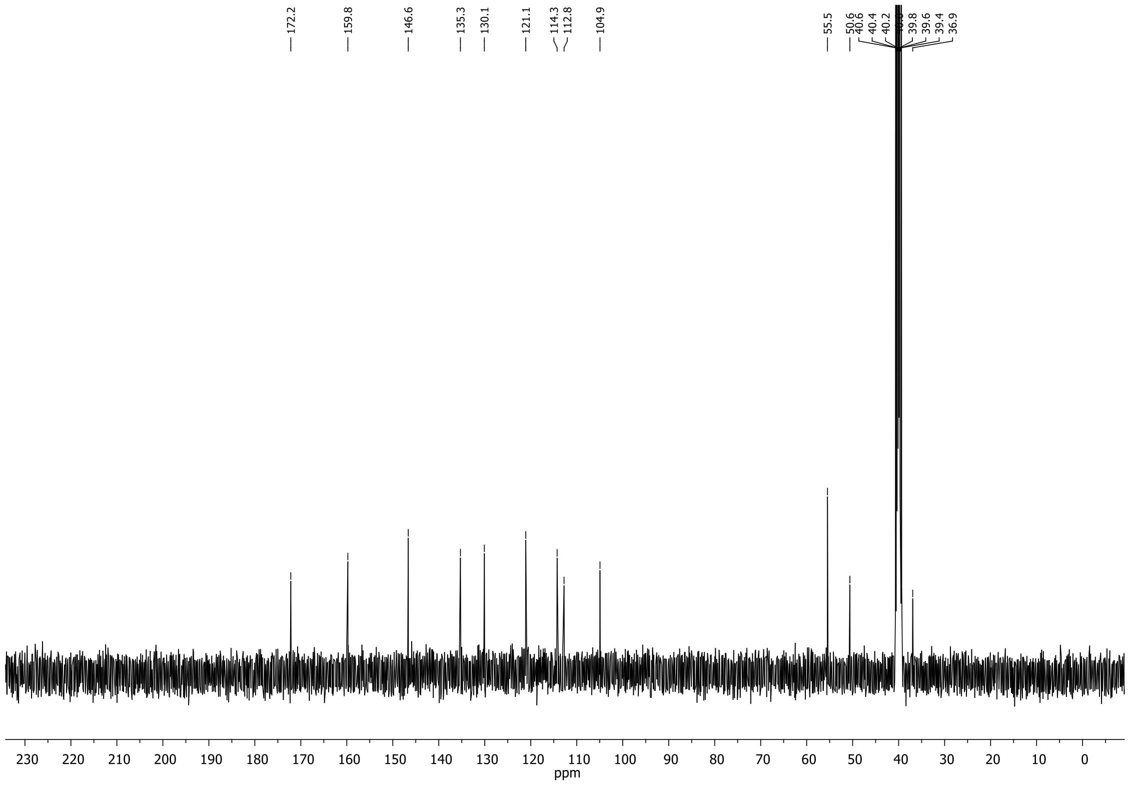

Figure S16. ^{13}C NMR of 3,8-bis(3-methoxybenzylidene)-2,7-dioxaspiro[4.4]nonane-1,6-dione (**5h**).

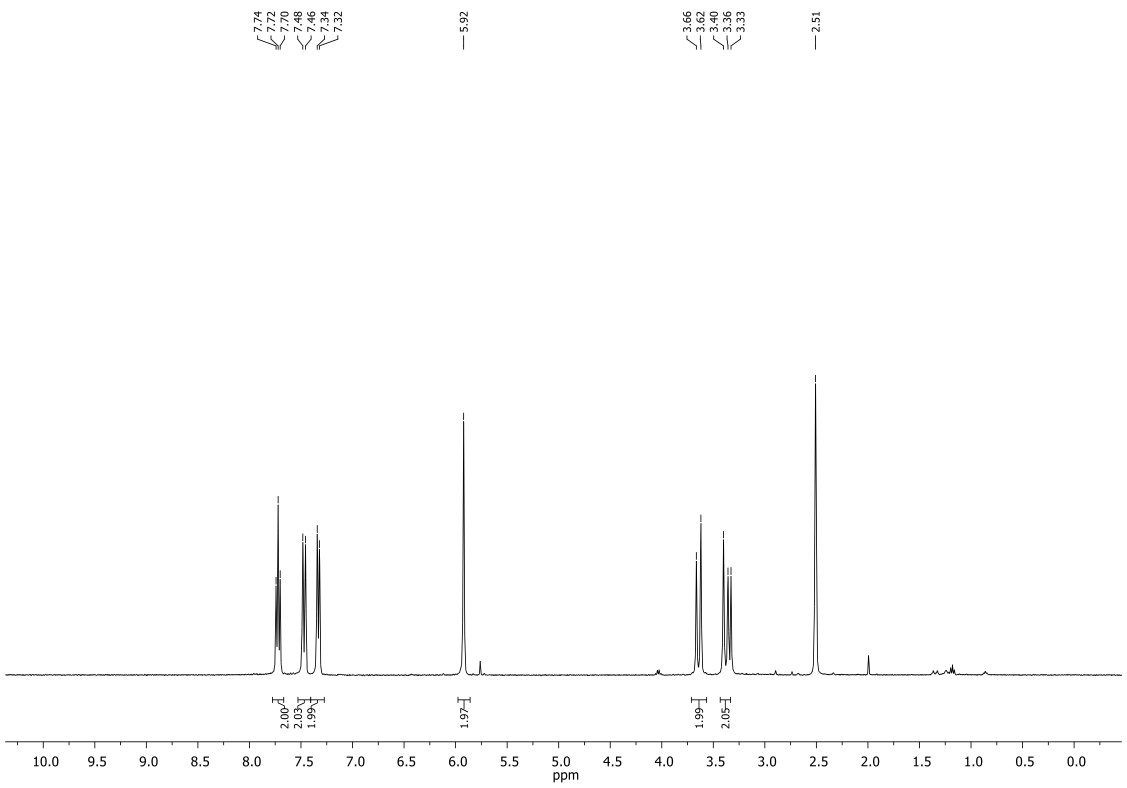

Figure S17. ^{1}H NMR of 3,8-bis(4-bromo-3-fluorobenzylidene)-2,7-dioxaspiro[4.4]nonane-1,6-dione (**5i**).

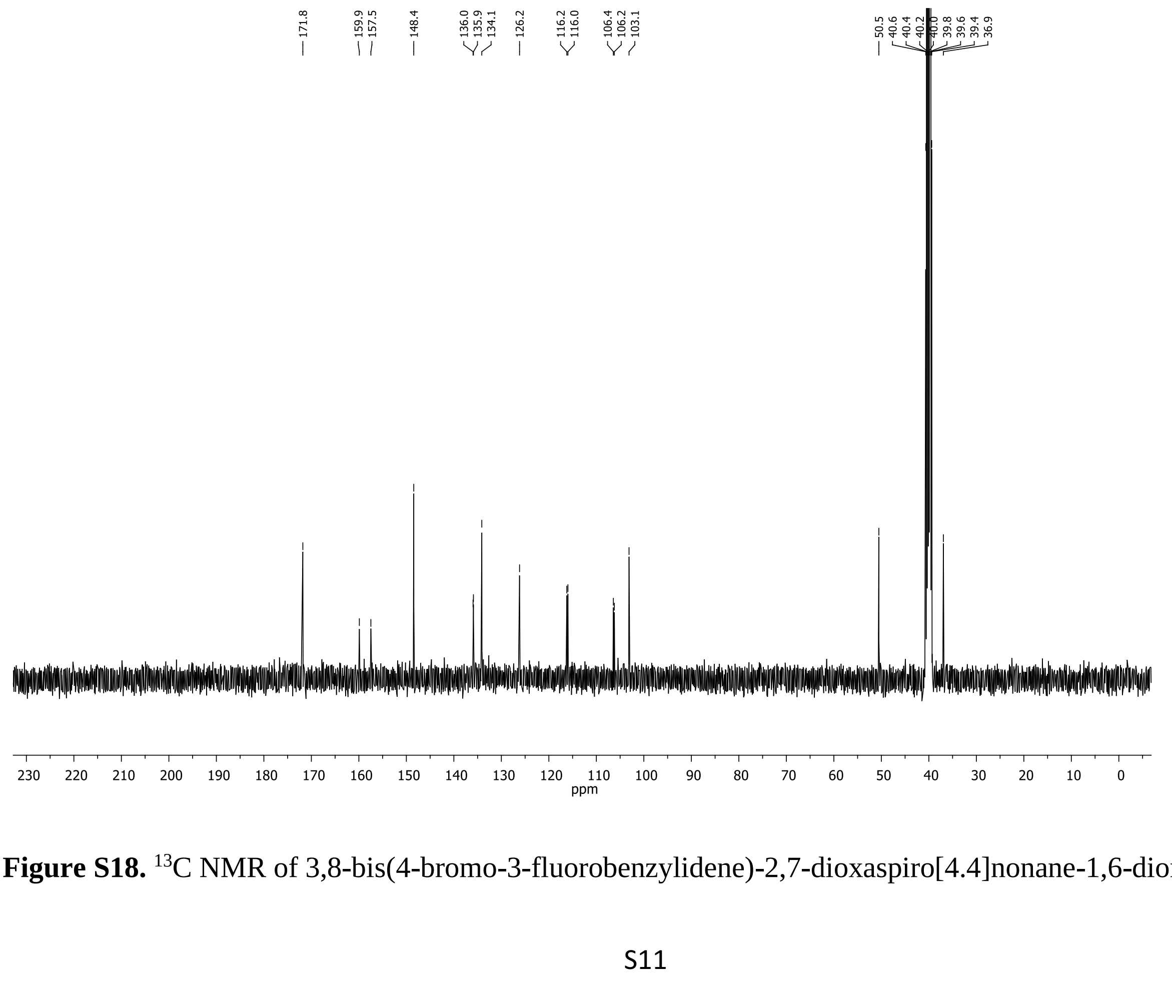

Figure S18. ^{13}C NMR of 3,8-bis(4-bromo-3-fluorobenzylidene)-2,7-dioxaspiro[4.4]nonane-1,6-dione (**5i**).

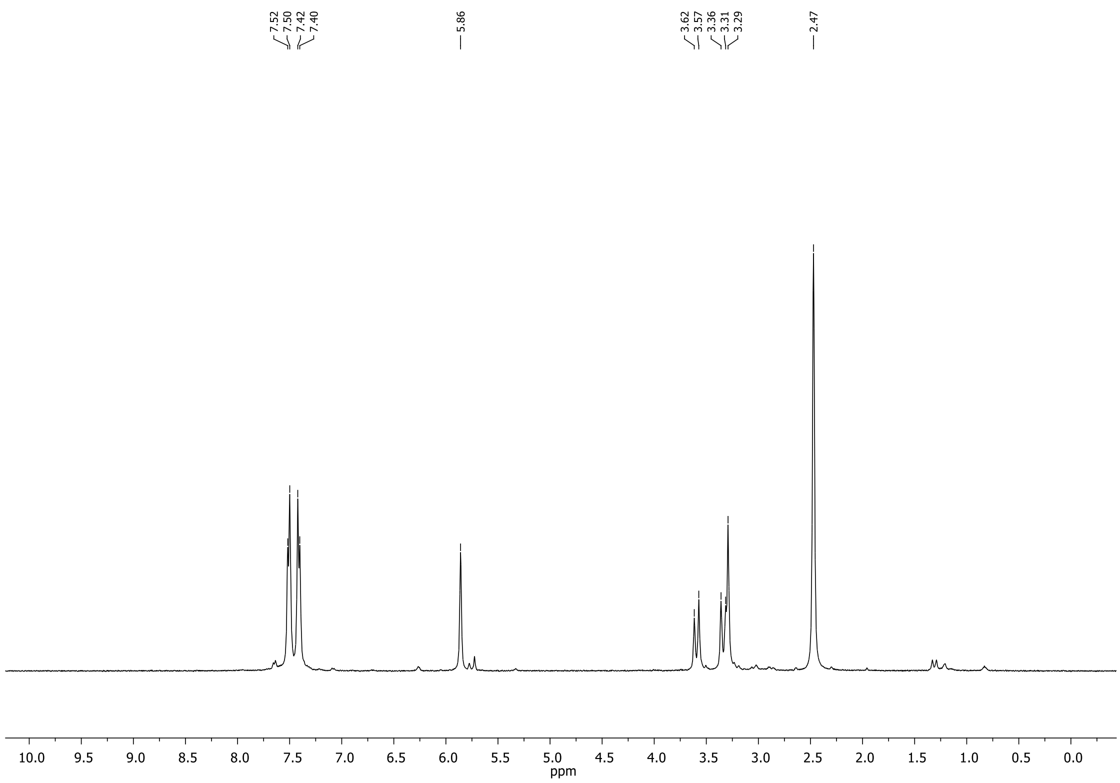

Figure S19. ^{1}H NMR of 3,8-bis(4-chlorobenzylidene)-2,7-dioxaspiro[4.4]nonane-1,6-dione (**5j**).

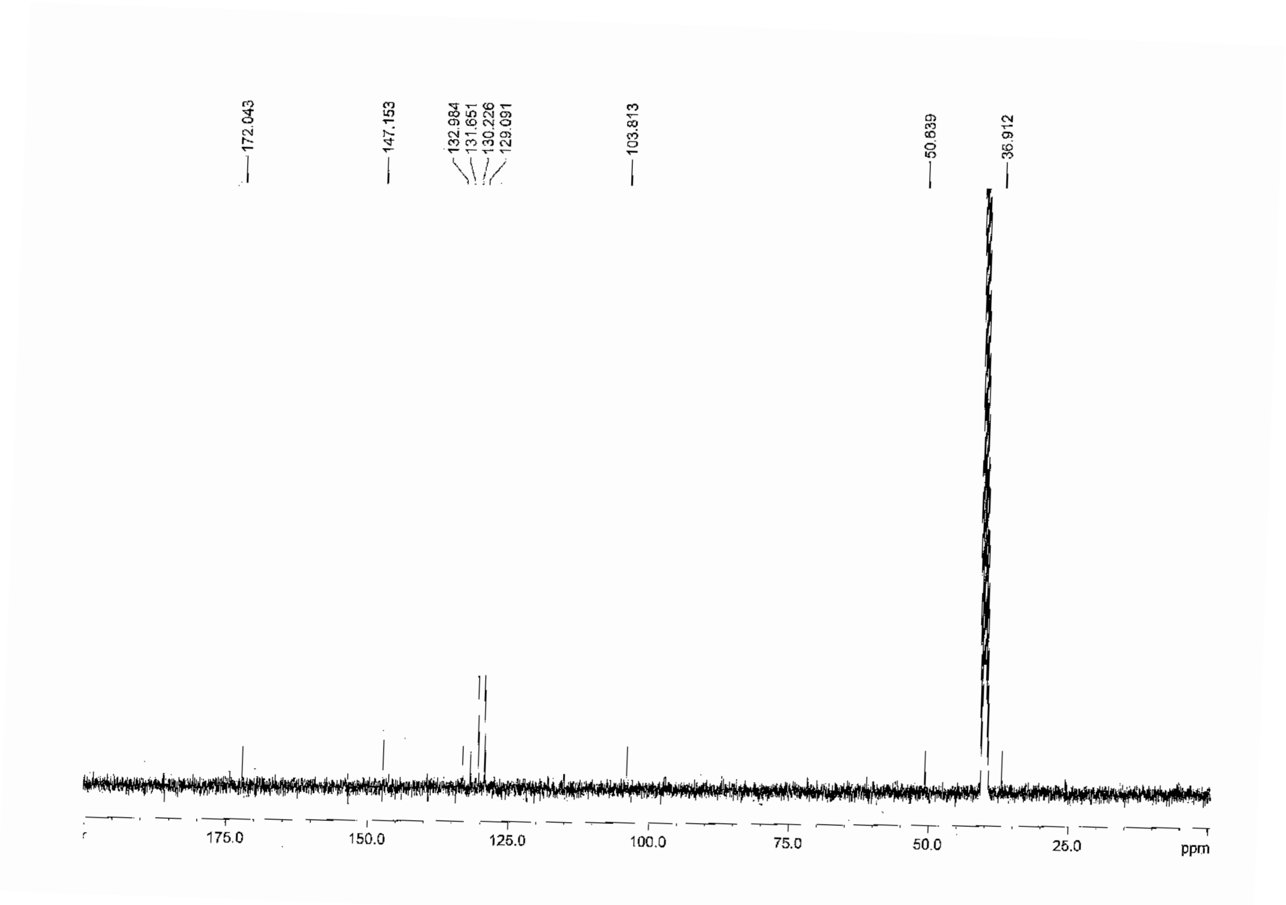

Figure S20. ^{13}C NMR of 3,8-bis(4-chlorobenzylidene)-2,7-dioxaspiro[4.4]nonane-1,6-dione (**5j**).

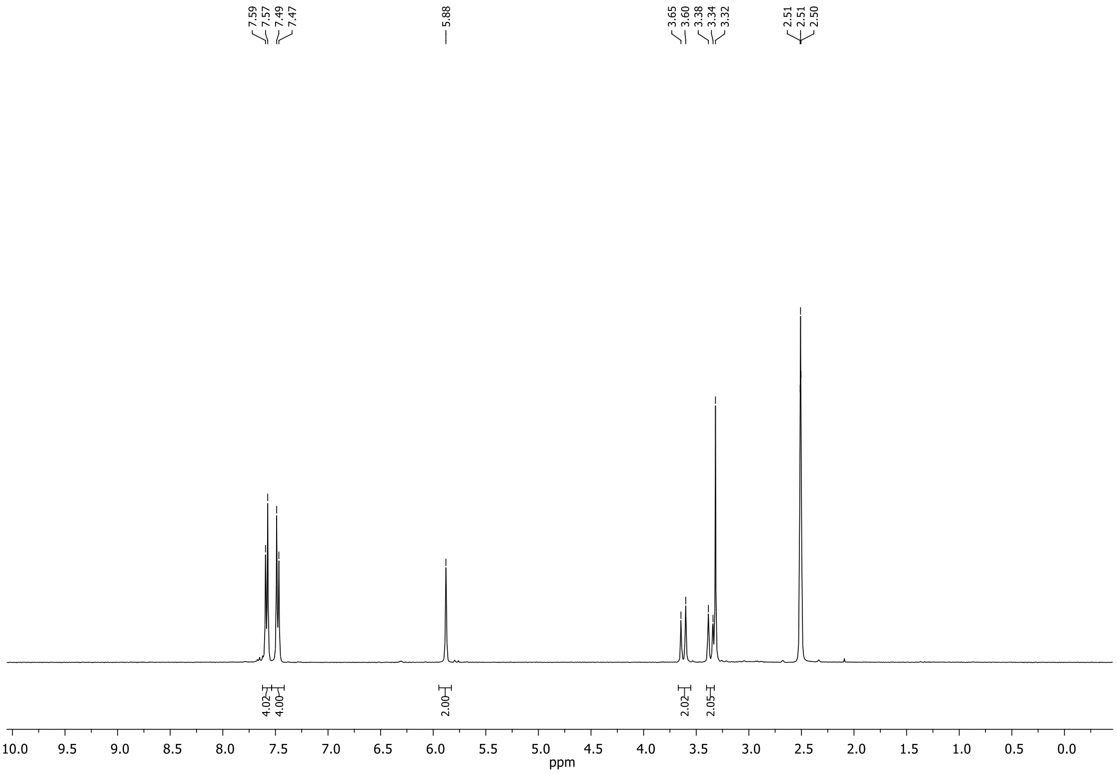

Figure S21. ^{1}H NMR of 3,8-bis(4-bromobenzylidene)-2,7-dioxaspiro[4.4]nonane-1,6-dione (**5k**).

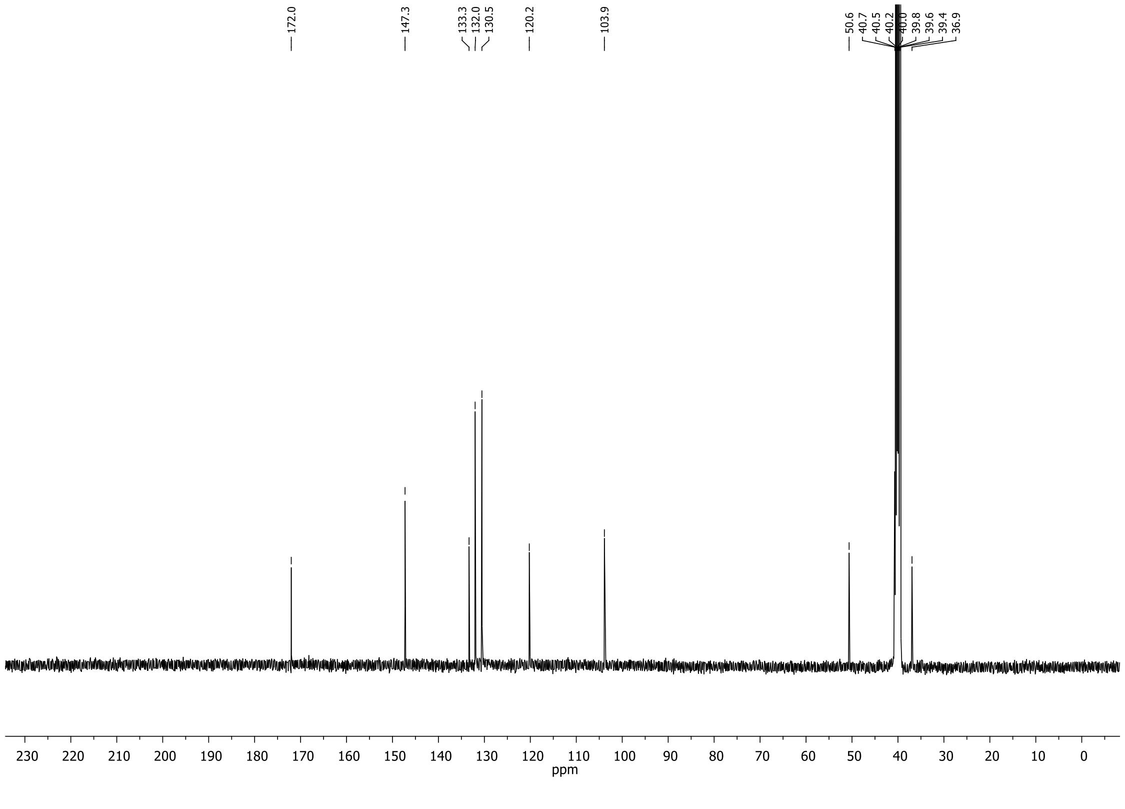

Figure S22. ^{13}C NMR of 3,8-bis(4-bromobenzylidene)-2,7-dioxaspiro[4.4]nonane-1,6-dione (**5k**).

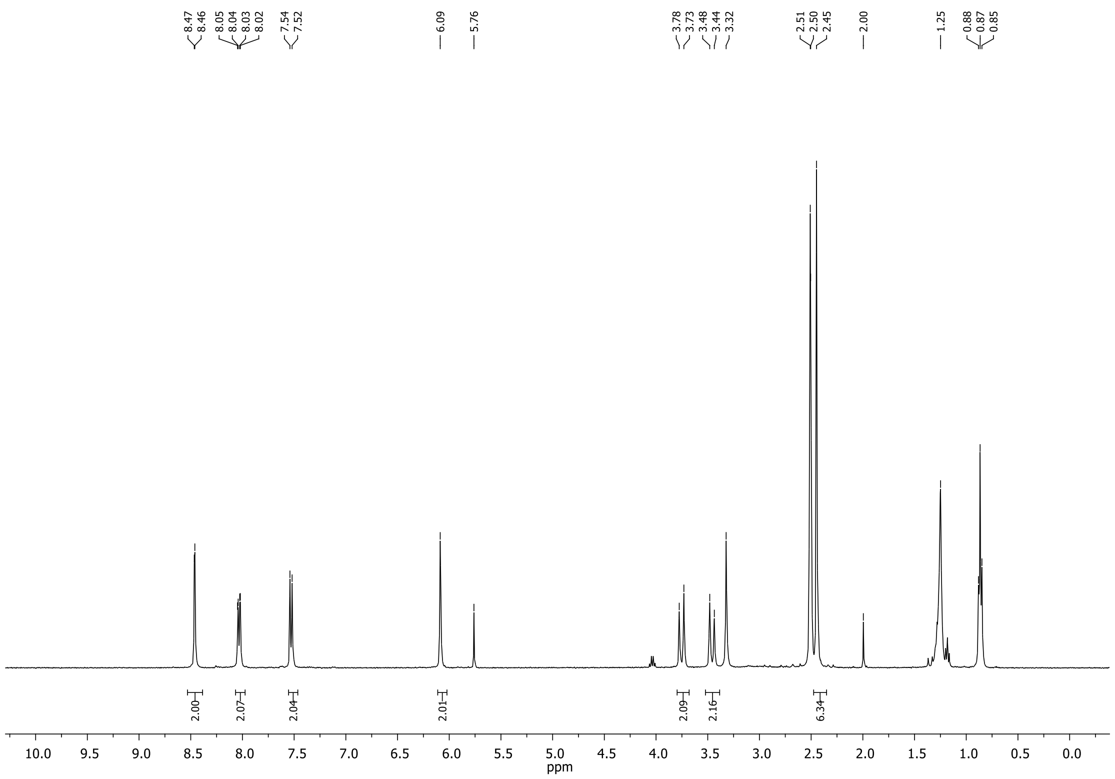

Figure S23. ^{1}H NMR of 3,8-bis(2-methyl-5-nitrobenzylidene)-2,7-dioxaspiro[4.4]nonane-1,6-dione (**5l**).

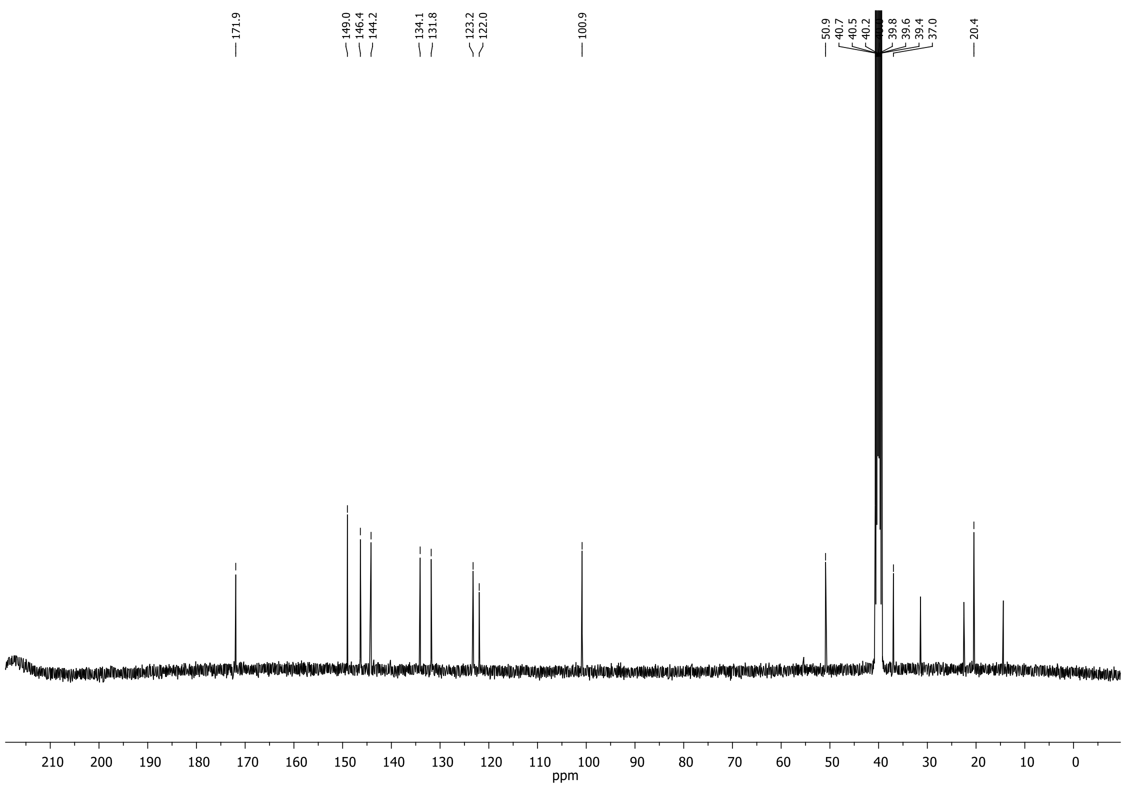

Figure S24. ^{13}C NMR of 3,8-bis(2-methyl-5-nitrobenzylidene)-2,7-dioxaspiro[4.4]nonane-1,6-dione (**5l**).

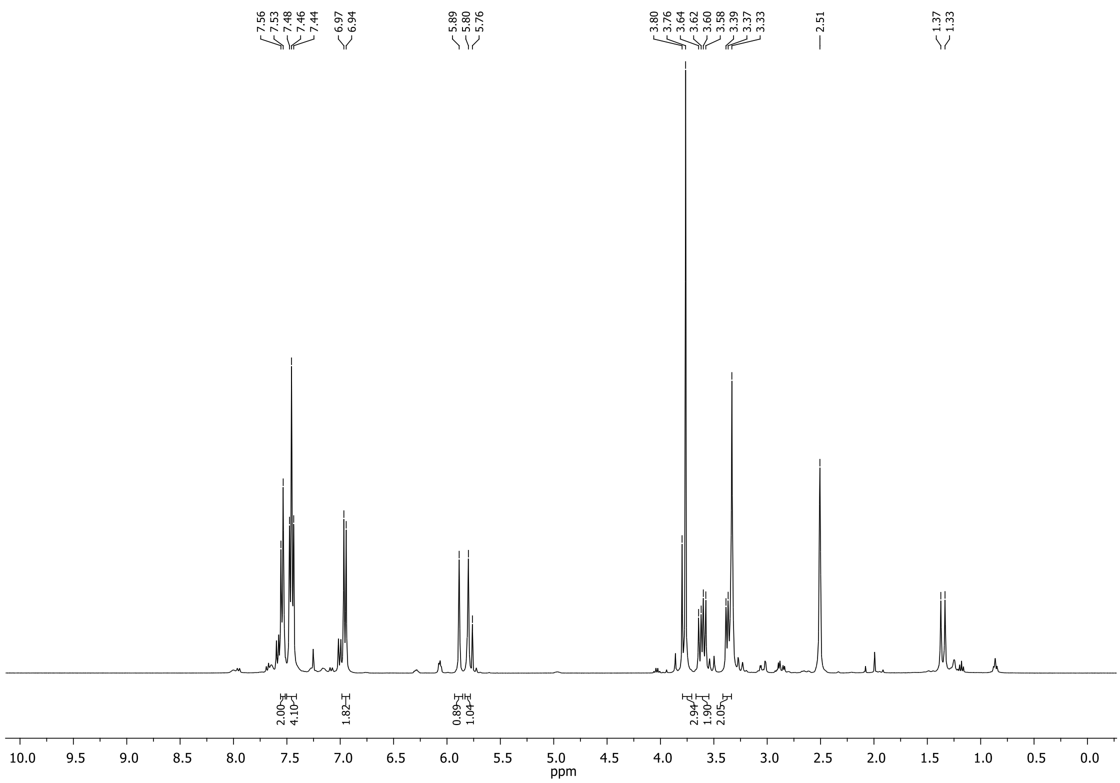

Figure S25. ^{1}H NMR of 3-(4-chlorobenzylidene)-8-(4-methoxybenzylidene)-2,7-dioxaspiro[4.4]nonane-1,6-dione (**5m**).

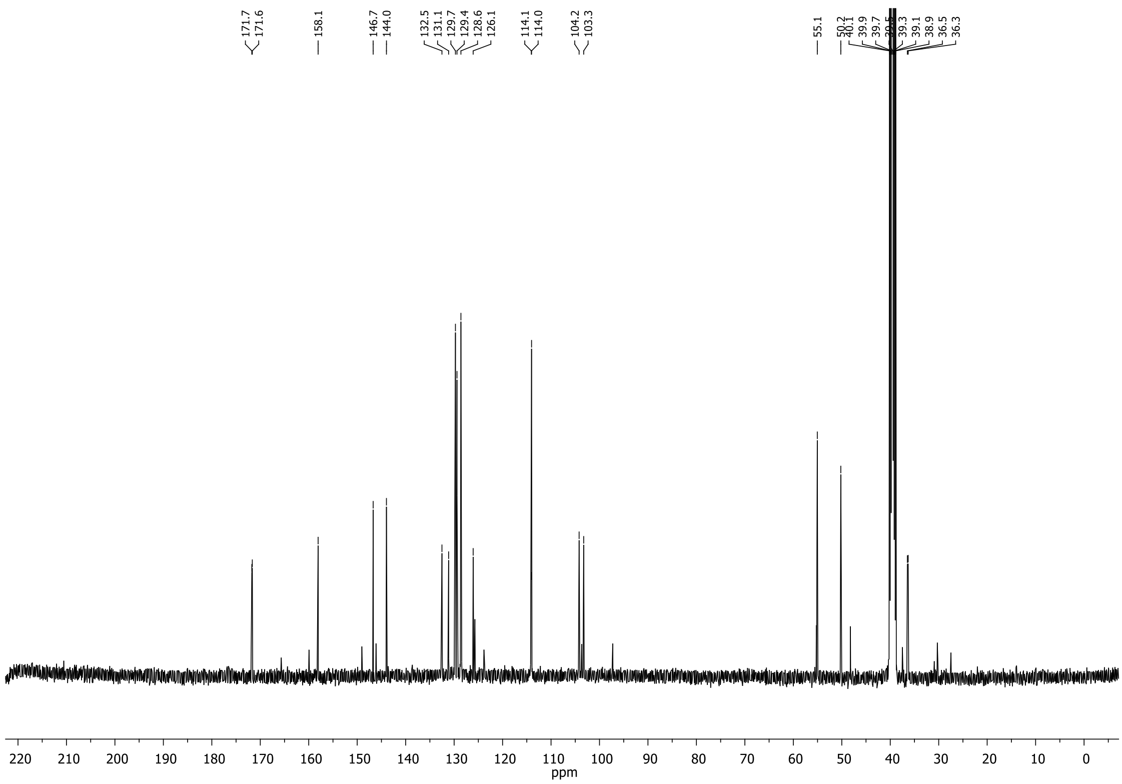

Figure S26. ^{13}C NMR of 3-(4-chlorobenzylidene)-8-(4-methoxybenzylidene)-2,7-dioxaspiro[4.4]nonane-1,6-dione (**5m**).

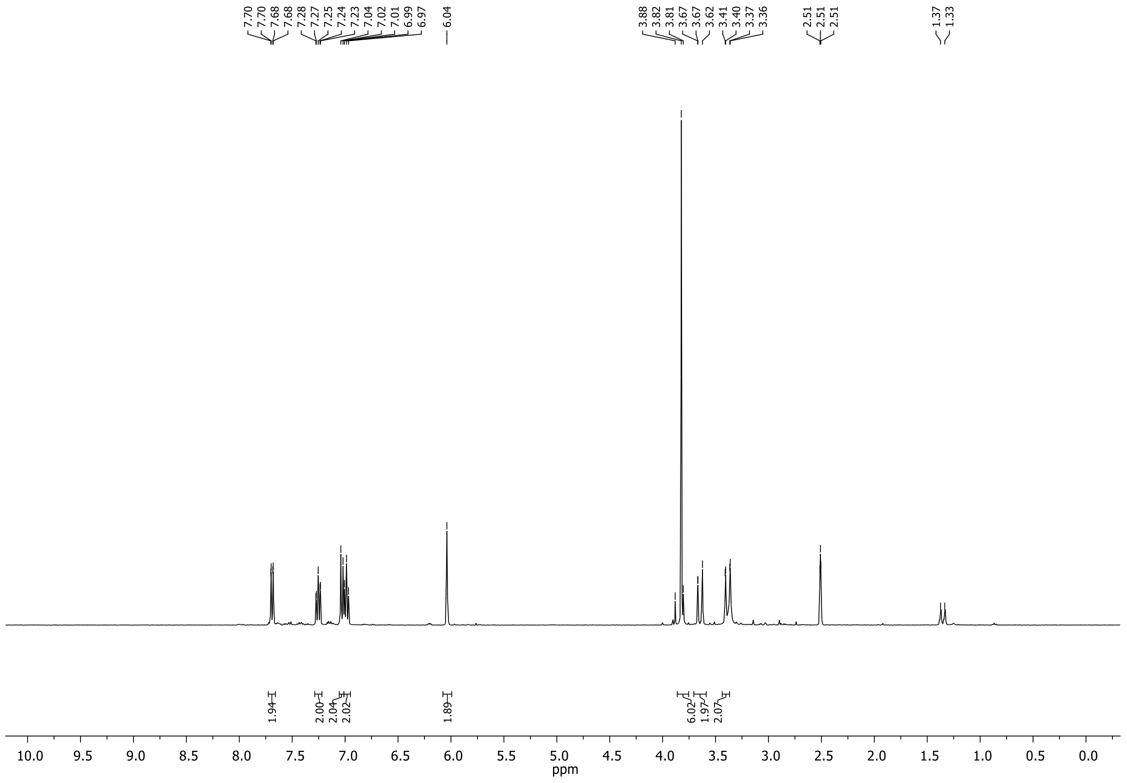

Figure S27. ^{1}H NMR of 3,8-bis(2-methoxybenzylidene)-2,7-dioxaspiro[4.4]nonane-1,6-dione (**5n**).

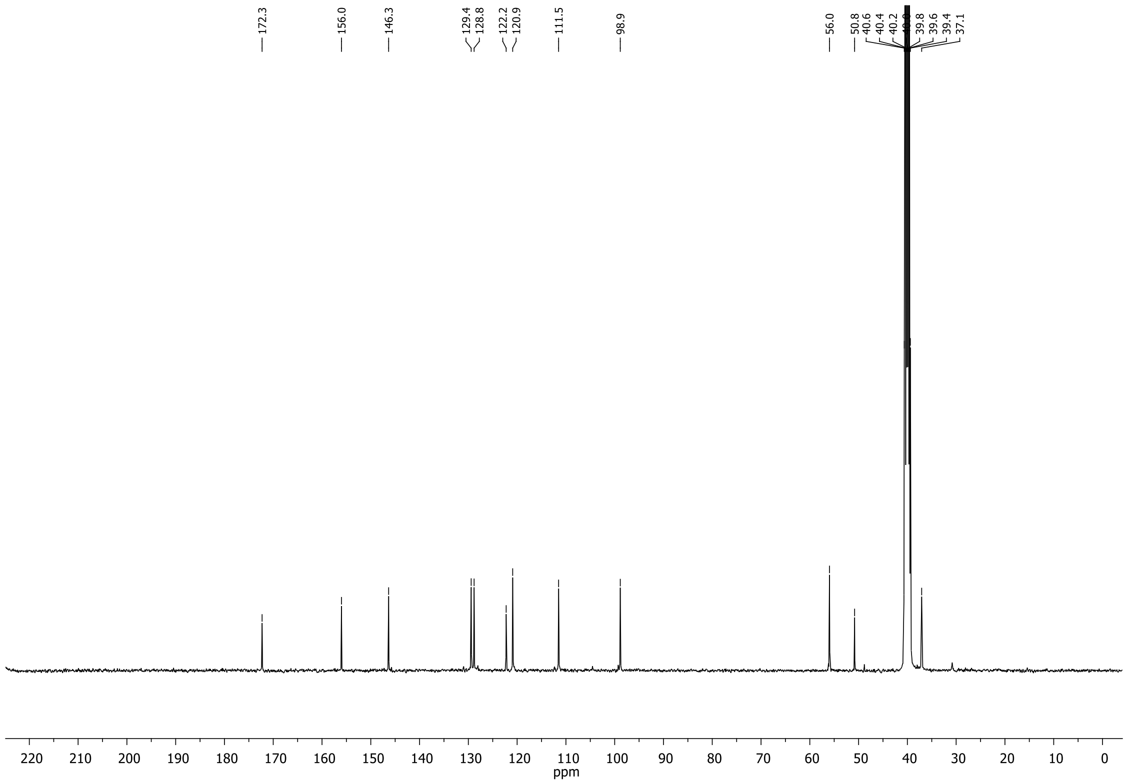

Figure S28. ^{13}C NMR of 3,8-bis(2-methoxybenzylidene)-2,7-dioxaspiro[4.4]nonane-1,6-dione (**5n**).
